# Injury-Inciting Activities in Male and Female Football Players: A Systematic Review

**DOI:** 10.1007/s40279-022-01753-5

**Published:** 2022-10-31

**Authors:** Francesco Aiello, Franco M. Impellizzeri, Susan J. Brown, Andreas Serner, Alan McCall

**Affiliations:** 1 Arsenal Performance and Research Team, Arsenal Football Club, London, UK; 2 School of Applied Sciences, Edinburgh Napier University, Edinburgh, UK; 3 Faculty of Health, Sport and Exercise Discipline Group, University of Technology Sydney, Sydney, Australia; 4 FIFA Medical, Fédération Internationale de Football Association, Zurich, Switzerland

## Abstract

**Background:**

A comprehensive examination of the sport-specific activities performed around the time of injury is important to hypothesise injury mechanisms, develop prevention strategies, improve management, and inform future investigations. The aim of this systematic review is to summarise the current literature describing the activities performed around the time of injury in football (soccer).

**Methods:**

A systematic search was carried out in PubMed, Web of Science, SPORTDiscus, and OpenGrey. Studies were included if participants were football players aged > 13 years old and the activities performed at the time of injury were reported together with the total number of injuries. Risk of bias was assessed using an adapted version of checklists developed for prevalence studies. The activities reported by the studies were grouped to account for inconsistent reporting, and the proportion of each injury activity was calculated. Data were not meta-analysed due to high heterogeneity of methods and classification criteria.

**Results:**

We included 64 studies reporting on 56,740 injuries in total. ACL injures were analysed by 12 studies, ankle/foot and knee injuries were analysed by five studies, thigh injuries were analysed by four studies, hip/groin injuries were analysed by three studies, and hamstring injuries were analysed by two studies. Five studies analysed more than one type of injury and 38 studies did not specify the type of injuries analysed. Running and kicking were the predominant activities leading to thigh and hamstring injuries. Changing direction and kicking were the predominant activities leading to hip and groin injuries and duels were the predominant activities leading to ankle injuries. Duels and pressing seem the predominant activities leading to ACL injuries, while results for other knee and general injuries were inconsistent.

**Conclusions:**

A qualitative summary of the activities performed at the time of injury has been reported. The results need to be interpreted carefully due to the risk of bias observed in the included studies. If we are to meaningfully progress our knowledge in this area, it is paramount that future research uses consistent methods to record and classify injuries and activities leading up to and performed at the time of injury.

**Registration:**

The protocol of this systematic review was registered at the Open Science Framework (https://doi.org/10.17605/OSF.IO/U96KV).

**Supplementary Information:**

The online version contains supplementary material available at 10.1007/s40279-022-01753-5.

## Key Points

**Table:** 

High intensity running and kicking are reported as the most prevalent activities leading up to thigh and groin injuries.
Most of the studies reporting activity of injuries showed high risk of bias with the main limitation being methods implemented to report injuries and inciting activities.
The available literature does not allow for clear and reliable practical recommendations.
Future studies should define injuries using the most recent guidelines and should report the inciting activities using a standardised system which requires appropriate development.

## Introduction

Understanding how injuries occur is essential to develop meaningful preventive strategies [1]. The aetiology of football injuries is multi-factorial and many models have been developed to improve our understanding of internal and external risk factors that predispose players and make them more susceptible to injury [2]. A comprehensive understanding of the sport-specific activities performed before and at the time of injury is important for several reasons. For example, it can guide the development of playing rules that may help to reduce injury risk (e.g., changing the rules on the use of the upper extremity during heading to reduce risk of head injuries [3]) or the identification and selection of relevant activities to investigate from a biomechanical perspective in controlled conditions (e.g., studies evaluating the biomechanics of landing and impact on anterior cruciate ligament [ACL] injury risk [4, 5]). Furthermore, it can help researchers and practitioners to hypothesise potential causes and causal pathways that can be formally tested, and to eventually develop and examine efficacy of preventive interventions [1]. Finally, understanding whether there are more common activities performed around the time of injury can help in prioritising the areas of focus for interventions and investigations.

While systematic reviews on the biomechanics of injuries such as ACL [6, 7] and hamstring injuries [8] exist, no systematic review has been performed on the football-specific activities leading up to and performed at the time of injury. Therefore, the aim of this systematic review is to provide an overview of the activities leading up to and performed at the time of injury in football (soccer) at all levels in both males and females.

## Methods

The study protocol was developed following the guidelines provided by the Preferred Reporting Items for Systematic Reviews and Meta-analyses 2020 (PRISMA 2020) and A MeaSurement Tool to Assess systematic Reviews 2 (AMSTAR 2) [9, 10]. The protocol was first registered at the Open Science Framework in April 2020 and then updated in July 2020 (10.17605/OSF.IO/U96KV) with more detailed inclusion criteria for study selection.

### Terminology

Sport-specific activities performed before and at the time of injury are often described as *injury mechanisms* [11, 12]. However, this term may be confusing as it can also include the biomechanical description of the inciting event [11]. The term *mechanism* describes the mechanical deformations and physiologic responses that cause an anatomic lesion or functional change [13] and is used in epidemiology to describe the scenario which includes all of the components which contribute to the sufficient cause for a certain outcome [14]. Therefore, *injury mechanism* may not be the most appropriate term to refer to the sport-specific activities performed before and at the time of injury. *Injury circumstance* is a term that indicates the environmental factors surrounding an injury, such as the activity performed by the injured person and the time and place of injury [15–17]. The term was developed with a focus on health and safety, and not sports injuries, and therefore there is no consensus on how to define the sport-specific activity performed by the injured player around the time of injury. We acknowledge that the injuries would not be caused by the activity per se but by the sum of the mechanical forces occurring during such activity which exceeds the load tolerance of the tissue, and we therefore propose the term *(injury-) inciting activity* defined as the sport-specific activity during which injuries occur, and the term *(injury-) inciting circumstance* defined as the environmental factors which surround an injury (including several aspects of the injury such as the inciting activity, playing phase, and pitch position).

### Eligibility Criteria

The following inclusion criteria were used to select the studies: peer-reviewed articles and grey literature written in English and reporting on competitive football players (any level) age > 13 years old; number of injuries; inciting activities during which lower limb injuries occurred. Studies were excluded if they were reviews, meta-analyses, opinion pieces, and reports with abstract only, as well as studies involving recreational players, elderly, military, and clinical populations because they may have used different playing rules.

### Search Strategy

A systematic search was carried out in PubMed (MEDLINE), Web of Science, SPORTDiscus (EBSCO) and Open Grey to include articles from inception to April 2020. Search criteria were based on the Population, phenomena of Interest, Context (PICo) framework [18] as follows: population was considered as football players; phenomena of interest were considered as inciting activities in which injuries occurred; context was considered as any match or training session. The following search strategy was used for all the databases: (football OR soccer) AND (injur* AND (mechanism* OR event* OR situation* OR circumstance* OR occasion* OR activit* OR characteristic*)) AND (training OR match* OR game* OR competition*) (Table S1, see electronic supplementary material [ESM]). A second search was performed after completion of data extraction (July 2020) to ensure the inclusion of the most recent studies that may have not been available previously. Additionally, to ensure the inclusion of all relevant studies, further studies have been searched by consulting research experts, hand searching, and checking the references of articles obtained in the original search. After the completion of the full-text selection the web source Connected Papers (https://www.connectedpapers.com/) was used to find additional relevant studies. Finally, an updated search was performed in December 2021 to ensure the inclusion of the most recent studies prior to analysis.

### Study Selection

Independent screening of titles and abstracts of the studies was performed by two researchers (FA and FMI) using Endnote X9.3.3 (Clavirate, Philadelphia, USA). One reviewer had limited experience and one had extensive experience (> 10 publications) and formal education training in systematic review and meta-analysis. A comprehensive selection was applied as authors believed that inciting activities are also reported as additional analyses in the full texts. To be included for further assessment, titles had to specify a football or soccer population and to include the word “injury” or a synonym thereof, while abstracts had to report any information relating to injuries, such as incidence or severity. Subsequently, one reviewer (FA) screened the full texts, and another reviewer (FMI) checked a random selection of the included studies (52%) and all the full texts with initial inclusion uncertainty. Studies reporting data from a mixed population (e.g., various sports and ages) were included if it was possible to extract data for the specific population of interest. To evaluate inter-rater reliability of the inclusion process, Cohen’s kappa coefficient between the two reviewers was separately calculated for selection of abstracts and full texts [19]. Disagreements between the two reviewers were solved by a third reviewer (AM, with experience in systematic reviews and meta-analyses).

### Data Extraction

Data regarding sample size, player characteristics, aim of the studies, results, and methods implemented for injury data collection and analysis were extracted by one reviewer (FA), and a random sample was verified by a second reviewer (AM) for accuracy. The percentage of agreement was calculated to evaluate inter-rater agreement. When needed, data from figures were obtained using a validated web-based app (WebPlotDigitizer V4.3, https://automeris.io/WebPlotDigitizer, Pacifica, California, USA) [20]. The percentage of injuries which occurred during each inciting activity was obtained from each study if it was reported, otherwise it was determined by calculating the ratio between the number of injuries that occurred during each inciting activity and the total number of injuries that occurred. When both general (e.g., contact with another player) and detailed inciting activities (e.g., tackling and being tackled) were reported, only the detailed activities were analysed.

During the extraction process we found that similar inciting activities were described differently. We therefore agreed on merging similar inciting activities into general categorisations to facilitate the analysis (Table 1). Data concerning playing phases and pitch position at time of injury were not merged and are presented as originally reported in the studies.

**Table 1:** Categorisation of injury-inciting activities

Merged category	Activity as originally reported by the included studies
Ball handling	Ball handling/dribbling Ball handling/controlling Ball recovery Ball possession Ball protection Chasing a loose ball Dribbling/shielding Dribbling Pass cutting Passing/receiving pass Regaining balance after reaching Reaching Receiving pass/blocking shot Receiving Stretching
Blocking	Blocking Blocking a shot or pass Deflection
Changing direction	Changing direction Cutting Running, intention of turning Twisting/turning
Duel	Contact/collision Collision Player–player contact (excluding slide tackle) Contact with another player Direct trauma Duel Foul Impact Stepped on/fallen/kicked Kicked Tackling/being tackled Slide tackle Receiving a charge Kick/knee from opponents Tackled Tackling Violent conduct Use of elbow Heading Pressing Stepped on
General running	Distorting Falling Lateral movements Lunging Planting Running or other individual activities Running Running/jumping Slipping Tilting
High intensity running	Accelerating Acceleration/cutting Conditioning Decelerating Running/sprint Sprinting/high-speed running Sprinting
Jumping	Jumping/landing Jumping Landing
Kicking	Clearing Crossing Regaining balance after kicking Shooting/kicking Kicking Passing/shooting Passing Shooting
Other activities	Artificial turf Playing field conditions Turf Weather conditions
Unspecified activities	Attacking ball/opponent Ball non-possession Defending General play Set pieces Specific action Technique Throwing

### Assessment of Risk of Bias

We selected the tool for the assessment of risk of bias (RoB) based on the included studies. We modified a validated assessment of RoB tool for prevalence studies developed by Hoy et al. [21] to ensure the RoB tool addressed elements related to studies on inciting activities in football.

We excluded two items from the previous checklist (items 2 and 3). Item 2 evaluates whether the sampling frame was a true or close representation of the target population, and item 3 evaluates whether some sort of random selection is used to select the sample. Since sampling frames were not used by the included studies, these items were not deemed applicable and therefore removed. Furthermore, we believed that descriptions of study samples should be as accurate as possible to understand what population these results may be applied to, therefore to evaluate this aspect, we included one additional item from the NIH Quality Assessment Tool for Observational Cohort and Cross-Sectional Studies [22]. The final RoB tool included the following items: (1) study population representativeness of the target population; (2) description of the study participants; (3) how missing data were dealt with; (4) data collection methods; (5) injury definition; (6) instruments for the measurement of the parameter of interest; (7) homogeneity of data collection methods; (8) length of shortest prevalence period; (9) reporting of numerator(s) and denominator(s) for the parameter of interest. A complete explanation of the adapted tool and of the criteria used to evaluate the RoB is available in Table S2 (see ESM).

The RoB of the included studies was assessed scoring each item as ‘Yes’, ‘Partially’, or ‘No’ if all, some, or none of the scoring criteria were respected. When information was not clear enough to score an item, it was rated as ‘Unclear’, while items deemed as not applicable were rated as such. Items scored as ‘Unclear’ and ‘No’ were rated as ‘high RoB’, items scored as ‘Partially’ were rated as ‘medium RoB’, and items scored as ‘Yes’ were rated as ‘low RoB’. Not applicable items were not included in the rating.

The scoring criteria were discussed and refined by two researchers (FA and FMI) until almost perfect agreement was reached (*K* = 0.87) (Table S3 in the ESM). The same reviewers screened 52% (*n* = 33) of the studies included and one reviewer (FA) scored the remaining studies. Each domain was assessed individually, and an overall rate of each domain was calculated, without trying to collate an overall score for each study [10]. Any disagreements between the two reviewers were resolved by discussion and when agreement was not achieved, a final decision was taken by a third reviewer (AM).

### Data Analysis

Data were presented descriptively with the inciting activities reported in tables and the proportion of the inciting activity categories in figures. Due to high data heterogeneity of the studies, performing a meta-analysis was not possible.

Data were analysed using RStudio version 1.3.1056 and packages *reshape2* and *ggplot2* [23–25], and an interactive dashboard was created with Tableau 2021.1 to allow the reader to explore the inciting activities according to injury location and by player characteristics reported by the included studies (playing level and sex).

## Results

### Search Results

**Fig. 1:**
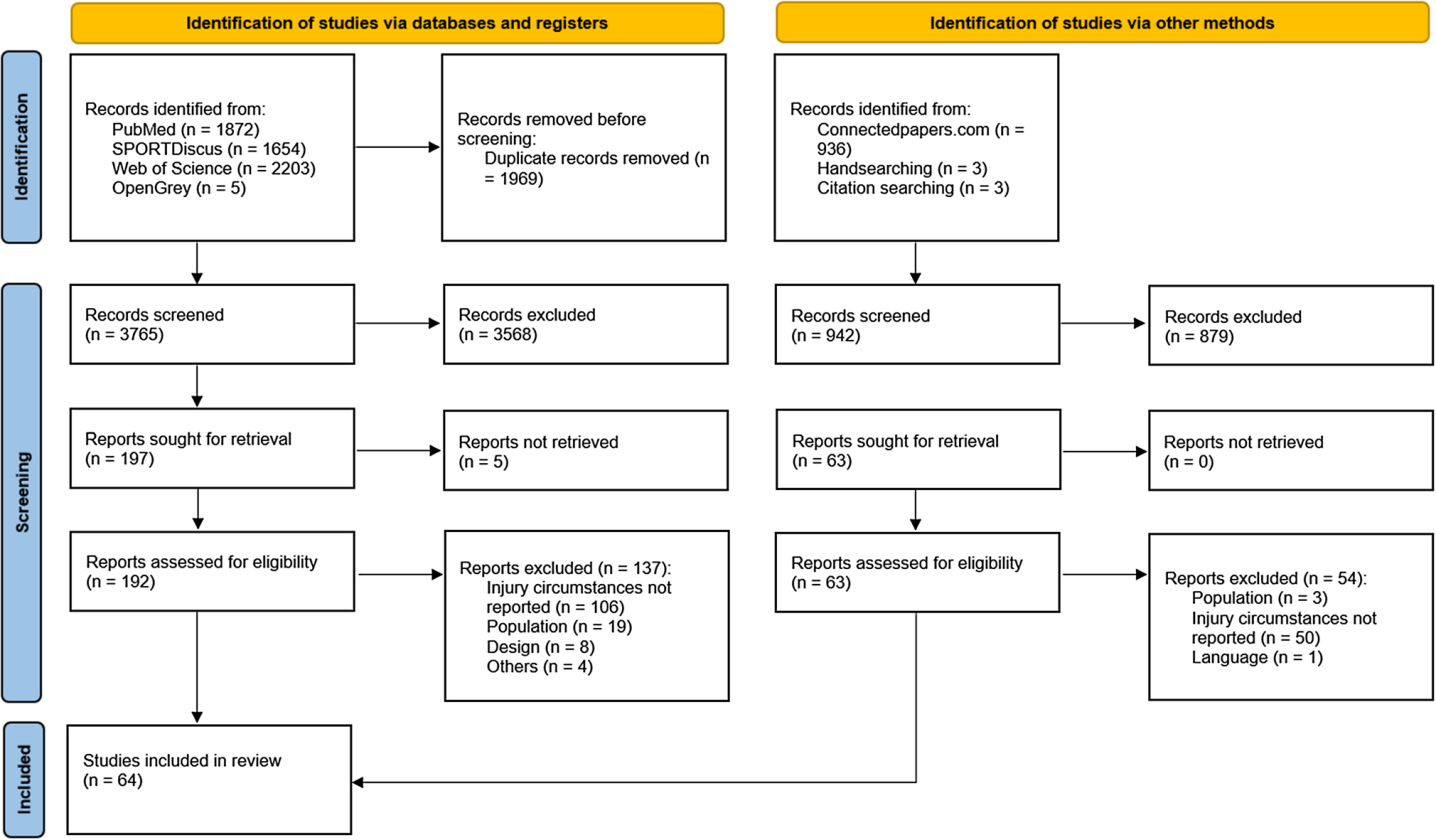
PRISMA 2020 flow diagram for new systematic reviews which included searches of databases, registers, and other sources

The systematic search provided 5734 articles, of which 1969 were duplicates, leaving 3765 for screening. After abstract selection, 197 full texts were screened, 142 were excluded (Table S4, see ESM), and 55 were deemed eligible to be included in the review. Five additional studies were retrieved by reference checking and hand searching, and four were retrieved from Connected Papers. In total, 64 studies were included in the systematic review, all of which were peer-reviewed (Fig. 1). Substantial agreement was shown in titles and abstract screening (*K* = 0.69; percentage agreement = 96%), and perfect agreement was shown in full-text selection (*K* = 1; percentage agreement = 100%). We extracted data from five studies [26–30] using WebPlotDigitizer V4.3, and 87% agreement was shown for data extraction. The PRISMA checklist is reported in Table S5 (see ESM).

### Study Characteristics

Male participants only were included in 43 studies, females only in 13 studies, and both males and females in eight studies. Players aged > 18 years old were included in 33 studies, players aged < 18 years old were included in nine studies, players of mixed ages were included in five studies and the remaining 17 studies did not clearly report the age of the players. Elite players were included in 10 studies and professional players were involved in 28 studies.

Twenty-eight studies did not aim to analyse inciting activities specifically, but nevertheless reported them in the results. This supports the comprehensive approach applied in the selection process. The method by which data on inciting activities were collected were medical reports filled out by Sports Medicine practitioners (32), video-based methods (20), questionnaires (12), and interviews (4). Sometimes more than one method was used to collect injury data. The injury definitions recommended for studies in football provided by Fuller et al. [31] were used by only 13 studies and only three of the 20 studies that analysed inciting activities with video analysis classified them using a standardised system. Inciting activities leading to general injuries were analysed by 38 studies (Table S6, see ESM), while other studies analysed the inciting activities leading to specific injuries such as ACL (12 studies) (Table 2), ankle and foot (5), knee (4), thigh (4), adductors/hip/groin (3), and hamstring (2) injuries (Table 3). Five studies reported more than one location/type of injury.

**Table 2:** Information from studies reporting inciting activities leading to ACL injuries

Study	Sex	Age (y)	Competitive level	Main aim	Tool	Injuries analysed (N)	Reported injury definition	Phase of play	Player location	Inciting activity (M–F)
Faunø and Wulff Jakobsen [32]	M	25.6, range 16–45	Elite and lower levels	Inciting activity analysis	Questionnaires	105	Unclear	Not reported	Opponents field: 59% Home field: 41% Opponents penalty box: 17% Home penalty box: 6%	Landing: 25% Turning: 63% Other*: 12%
Kaneko et al. [33]	F	17.4 ± 1.9	Not reported	Inciting activity analysis	Questionnaires	90	No	Attacking phase: 40% Defensive phase: 60%	Not reported	Clearing: 1% Cutting: 27% Dribbling: 16% Goalkeeping: 4% Heading: 3% Landing: 13% Loose ball: 7% Pass cutting: 4% Passing: 4% Pressing: 32% Shooting: 4% Sliding: 2% Stopping: 8% Trapping: 12%
Rochcongar et al. [34]	M	25.5 ± 2	Unclear	Inciting activity analysis	Questionnaires	611	Yes, not supported by any reference	Not reported	Not reported	Tackled: 12% Tackling: 3% Accelerating/cutting: 2% Jumping: 2% Kicking: 6% Landing: 21% Pivoting: 33%
Faude et al. [35]	F	22.4 ± 5.0	Professional	Other	Report	11	Yes, supported by consensus reference	Not reported	Not reported	Changing direction: 64% Foul play: 10% Tackling: 27%
Gupta et al. [36]	M & F	High school	High school	Other	Report	277	No	Not reported	Not reported	General play: 17–24% Chasing loose ball: 18–22% Defending: 16–21% Passing/shooting: 16–12% Ball handling/dribbling: 13–10% Receiving pass/blocking shot: 10–7% Heading: 0–2% Other*: 10–2%
Takahashi et al. [37]	M & F	M: 16.6 ± 0.9 F: 16.4 ± 0.9	High school	Inciting activity analysis	Report	100	No	Not reported	Not reported	Cutting and stopping: 13–21% Landing: 8–6% Other*:: 79–73%
Brophy et al. [38]	M & F	Adults and collegiate*	Professional and not	Inciting activity analysis	Video analysis	52	No	Ball possession: 27% No ball possession: 73%	Not reported	Tackled: 13% Tackling: 41–65% Cutting: 13–17% Dribbling: 3% Jumping: 3% Heading: 9% Kicking: 9–4% Receiving: 6–5% Running/jumping: 3–9%
De Carli et al. [39]	M	Adults*	Professional	Inciting activity analysis	Video analysis	128	No	Not reported	Offensive half: 63% Defensive half: 37%	Dribbling: 9% Passing: 6% Ball protection: 8% Shooting: 3% Stationary shooting: 1% Ball control: 8% Ball reception: 5% Ball recovery: 40% Other*: 20%
Della Villa et al. [40]	M	Adults*	Professional	Inciting activity analysis	Video analysis	134	No	Attacking phase: 32% Defensive phase: 68%	Defensive third: 37% Midfield third: 34% Offensive third: 29% Left side corridor: 25% Middle corridor: 50% Right side corridor: 25%	Tackled: 18% Tackling: 11% Cutting: 1% Diving: 1% Dribbling: 1% Jumping: 1% Regaining balance after kicking: 14% Landing: 6% Pressing: 30% Receiving: 2% Other*: 12%
Grassi et al. [41]	M	Adults*	Professional	Inciting activity analysis	Video analysis	34	No	Ball possession: 47% No ball possession: 53%	Opponents field: 68% Home field: 32%	Tackling: 15% Ball handling: 3% Crossing: 3% Defending: 9% Diving: 6% Dribbling: 18% Passing: 12% Pressing: 26% Running: 3% Shooting: 6%
Waldén et al. [42]	M	Adults*	Not reported	Inciting activity analysis	Video analysis	39	Yes, not supported by any reference	Attacking phase: 23% Defensive phase: 77%	Defensive middle: 21% Offensive middle: 23% Defensive third: 36% Offensive third: 21%	Collision: 10% Screening: 8% Tackled: 15% Clearing: 8% Diving: 3% Dribbling: 3% Heading: 13% Regaining balance after kicking:13% Passing: 3% Pressing: 28% Receiving: 5% Running: 10% Shooting: 3% Twisting/turning: 3%
Lucarno et al. [43]	F	Adults*	Professional	Inciting activity analysis	Video analysis	35	No	Attacking phase: 31% Defensive phase: 69%	Defensive third: 28% Midfield third: 37% Offensive third: 12% Left-side corridor: 20% Middle corridor: 40% Right corridor: 40%	Being tackled: 11% Pressing: 40% Tackling: 11% Landing: 3% Preparing to kick: 3% Regaining balance after kicking: 20%

Competitive levels are reported as originally described by the studies. Participants’ age is reported as originally described in the studies. If this was not described it was either deduced by the study or, when this was not possible, it was reported as unclear and indicated in this table with an asterisk (*)

Percentages are calculated by dividing the total number of injuries that occurred during each activity, phase of play, or location by the total number of injuries that occurred. Activities reported as ‘Other’ indicate the percentage of injuries whose activities were not reported or were reported as other or unknown

**Table 3:** Information from studies reporting inciting activities leading to other specific injuries

Study	Injury type	Sex	Age (y)	Competitive level	Main aim	Tool	Injuries analysed (N)	Reported injury definition	Phase of play	Player location	Inciting activity (M-F)
Serner et al. [44]	Adductors	M	27.5 ± 3.2	Elite	Inciting-activity analysis	Video analysis and interview	17	Unclear	Defensive phase: 53% Attacking phase: 41% No possession: 6%	Home penalty box: 24% Defensive third: 24% Mid-field third: 29% Offensive third: 12% Opponent penalty box: 12%	Changing direction: 35% Reaching: 24% Passing: 18% Jumping: 12% Shooting: 12%
Kofotolis et al. [45]	Ankle	M	24.8 ± 4.63	Amateur	Others	Questionnaire	139	Yes, supported by non-consensus reference	Not reported	Not reported	Contact with another player: 50% Landing: 9% Twisting/turning: 8% Running: 4% Falling: 2% Shooting: 2% Jumping: 2% Heading: 1% Diving: 1% Dribbling: 1% Passing: 1% Stretching: 1% Other*: 11%
Andersen et al. [46]	Ankle	M	Adults*	Professional	Inciting-activity analysis	Video analysis and questionnaire	26	Yes, not supported by any reference	Not reported	Not reported	Tackling: 15% Being tackled: 38% Clearing/shooting: 15% Running: 15% Landing: 8% Other*: 8
Giza et al. [47]	Ankle and foot	M	Adults, U20, U17*	Professional	Inciting-activity analysis	Video analysis	76	Yes, not supported by any reference	Not reported	Not reported	Tackler on his feet: 57% Tackler sliding: 37% Tackle from the side: 68% Tackle from behind: 24% Tackle from front: 8%
Krutsch et al. [48]	Ankle and knee	M	Adults*	Semi professional	Inciting-activity analysis	Video analysis	630	Yes, supported by non-consensus reference			Collision with teammate: 2% Collision with opponent: 12% Being kicked: 68% Being hit by opponent: 8% Tilting: 16% Distortion: 9% Blocking: 4% Sliding: 1% Overload: 4% Falling: 16%
Cross et al. [49]	Hamstring	M & F	Collegiate	College	Other	Report	519	Yes, not supported by any reference	Not reported	Defensive half: 50–60% Offensive half: 50–40%	Running: 69–71% Defending: 12–7% Passing/shooting: 10–12% Ball handling: 8–10%
Drummond et al. [50]	Hamstring	M	26.53 ± 4.75	Professional	Other	Report	92	No	Not reported	Not reported	Kicked: 6% Running/sprinting: 51% Kicking: 23% Jumping/landing: 6% Stretching: 3% Slipping: 3% Overuse 9%
Lundgårdh et al. [51]	Hip/groin	M	25 ± 5	Professional	Others	Report	467	Yes, supported by consensus reference	Not reported	Not reported	Change of direction: 6% Collision: 6% Jumping: 1% Landing: 1% Overuse: 27% Running: 6% Shooting: 15% Slipping: 2% Sprinting: 6% Stretching: 9% Other*: 20%
Ralston et al. [52]	Hip/groin	F	Collegiate	Collegiate	Other	Report	439	Yes, supported by consensus reference	Not reported	Not reported	General play: 44% Conditioning: 11% Diving: 9% Shooting: 8% Defending: 5% Passing: 4% Running: 4% Other*: 15%
Lundblad et al. [53]	Knee	M	Adults*	Professional	Inciting-activity analysis	Report	134	Yes, supported by consensus reference	Not reported	Not reported	Being tackled: 12% Collision: 6% Twisting/turning: 9% Being kicked: 1% Other*: 72%
Rahnama et al. [54]	Knee	M	23 ± 5.6	Professional	Other	Report and interview	43	No	Not reported	Not reported	Kicking: 27% Landing: 11% Pivoting and turning: 8% Falling: 2% Running: 2% Other*: 50
Buckthorpe et al. [55]	Knee	M	Adults*	Professional	Inciting-activity analysis	Video analysis	37	No	Defensive: 54% Offensive: 41%	Defensive third: 8% Midfield third: 46% Offensive third: 46% Left-side corridor: 6% Middle corridor: 57% Right-side corridor: 27%	Pressing/tackling: 38% Being tackled: 13% Other*: 51%
Nielsen and Yde [56]	Knee, ankle, foot, groin/thigh	M	Youths, adults	Unclear	Inciting-activity analysis	Report	109	Yes, not supported by any reference	Not reported	Not reported	Tackling: 40% Running: 39% Shooting: 6% Other*: 15%
Cross et al. [57]	Thigh	M & F	M: 16.14 + 1.22 F: 15.90 + 1.18	Amateur (high school)	Inciting-activity analysis	Report	350	Yes, not supported by any reference	Not reported	Defensive half: 41–7% Offensive half: 59–93%	Ball handling: 3–5% Defending: 11–6% Passing/shooting: 28–28% Running activities: 57–61% Other*: 4–5%
Ueblacker et al. [58]	Thigh	M	Adults*	Elite	Other	Report	2003	Yes, supported by consensus reference	Not reported	Not reported	Foul: 7% Shooting: 12% Sprinting/high-speed running: 53% Stretching: 3% Other*: 25%
Gronwald et al. [59]	Thigh	M	Adults*	Professional	Inciting-activity analysis	Video analysis	52	No	Not reported	Not reported	Acceleration: 27% High-speed running: 19% Kicking: 15% Landing: 4% Lunging: 31% Other*: 4%
Klein et al. [60]	Thigh	M	Adults*	Professional	Inciting-activity analysis	Video analysis	81	No	Not reported	Not reported	Sprinting: 43% Running: 23% Lunging:18% Other*: 16%

Competitive levels are reported as originally described by the studies. Participants’ age is reported as originally described in the studies. If this was not described it was either deduced by the study or, when this was not possible, it was reported as unclear and indicated in this table with an asterisk (*)

Percentages are calculated by dividing the total number of injuries that occurred during each activity, phase of play, or location by the total number of injuries that occurred. Activities reported as ‘Other’ indicate the percentage of injuries whose activities were not reported or were reported as other or unknown

### Inciting Activities (Global Perspective)

One hundred and seven different inciting activities were reported by the included studies, with the most frequently reported being tackling (reported by 38 studies), shooting (34), running (33), being tackled (29), heading (25), and passing (24). Additional information regarding the inciting circumstances were player location on the pitch (reported by 16 studies), playing phases (e.g., attacking or defensive phase, 15 studies), ball location (4), team action before the injury (2), and player focal attention (1).

The 64 studies included in the review reported inciting activities of 49,845 injuries in total (32,357 occurred in males, 17,488 occurred in females). Overall, 14,914 (30%) injuries occurred during duels, 12,846 (26%) during unspecified activities, 6376 (13%) during ball handling, 4089 (8%) during kicking, 3807 (8%) during general running, 1950 (4%) during high-intensity running, 1184 (2%) during changing direction, 1012 (2%) during jumping, 772 (1%) during blocking, and 39 (< 1%) for playing field conditions. Inciting activities of 2857 injuries (6%) were not described.

**Fig. 2:**
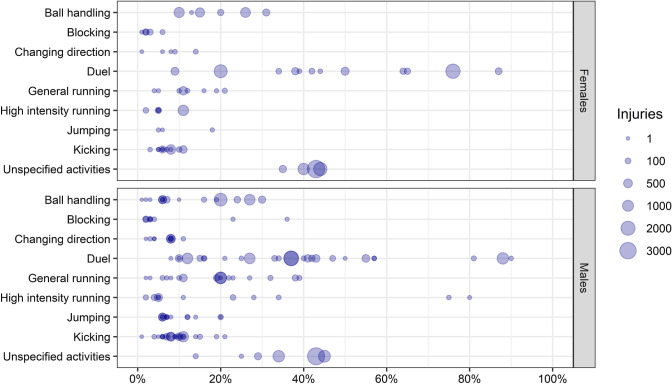
Percentage of injuries occurred during specific inciting activities by sex. Size of the dot represents the amount of injuries. Number of injuries reported: Females = 17,488, Males = 32,357

**Fig. 3:**
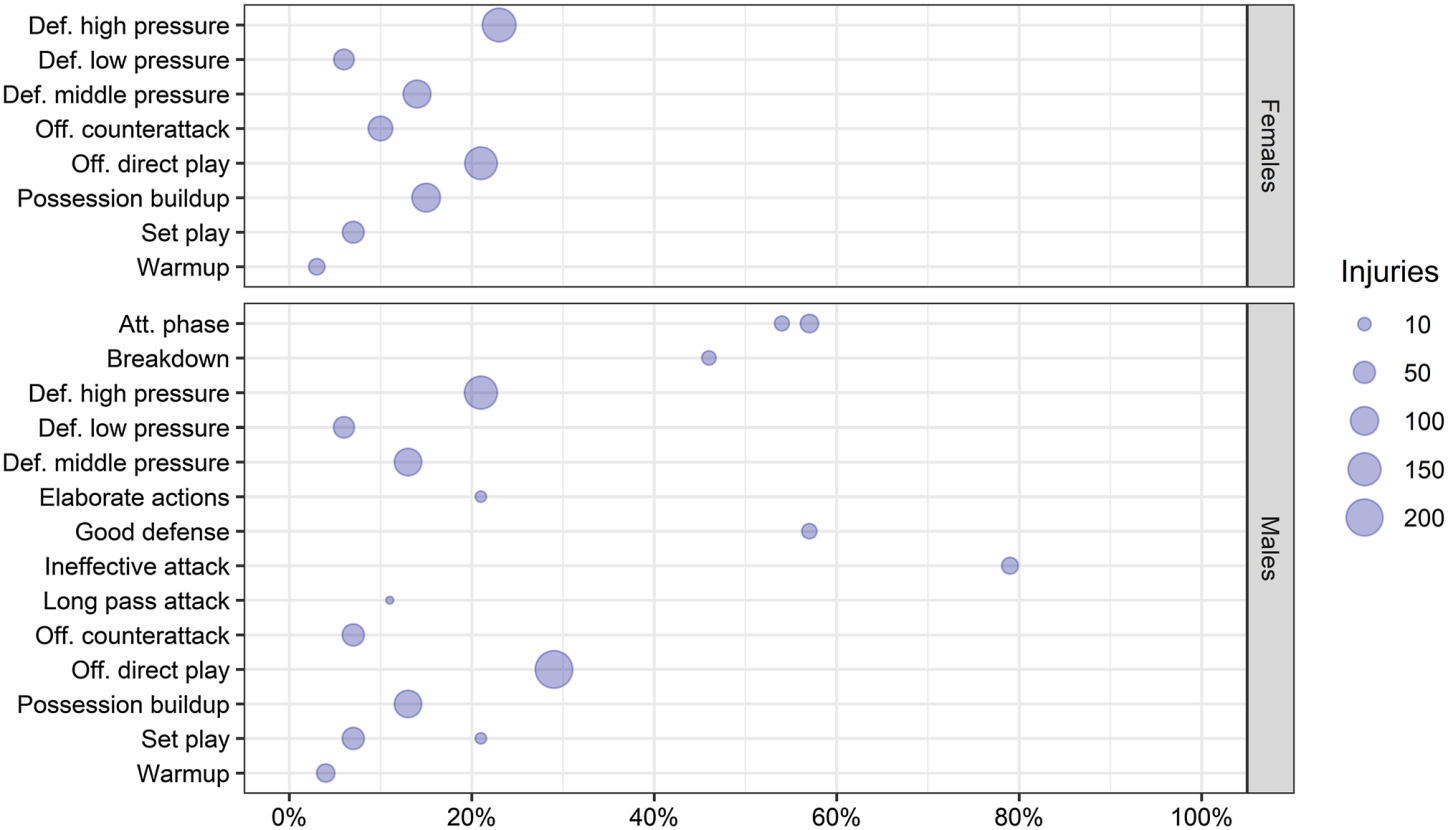
Percentage of injuries occurred in different playing phases by sex. Size of the dot represents the amount of injuries. Number of injuries reported: Males = 802, Females = 693. *Att.* attacking, *Def.* defensive, *Off.* offensive

**Fig. 4:**
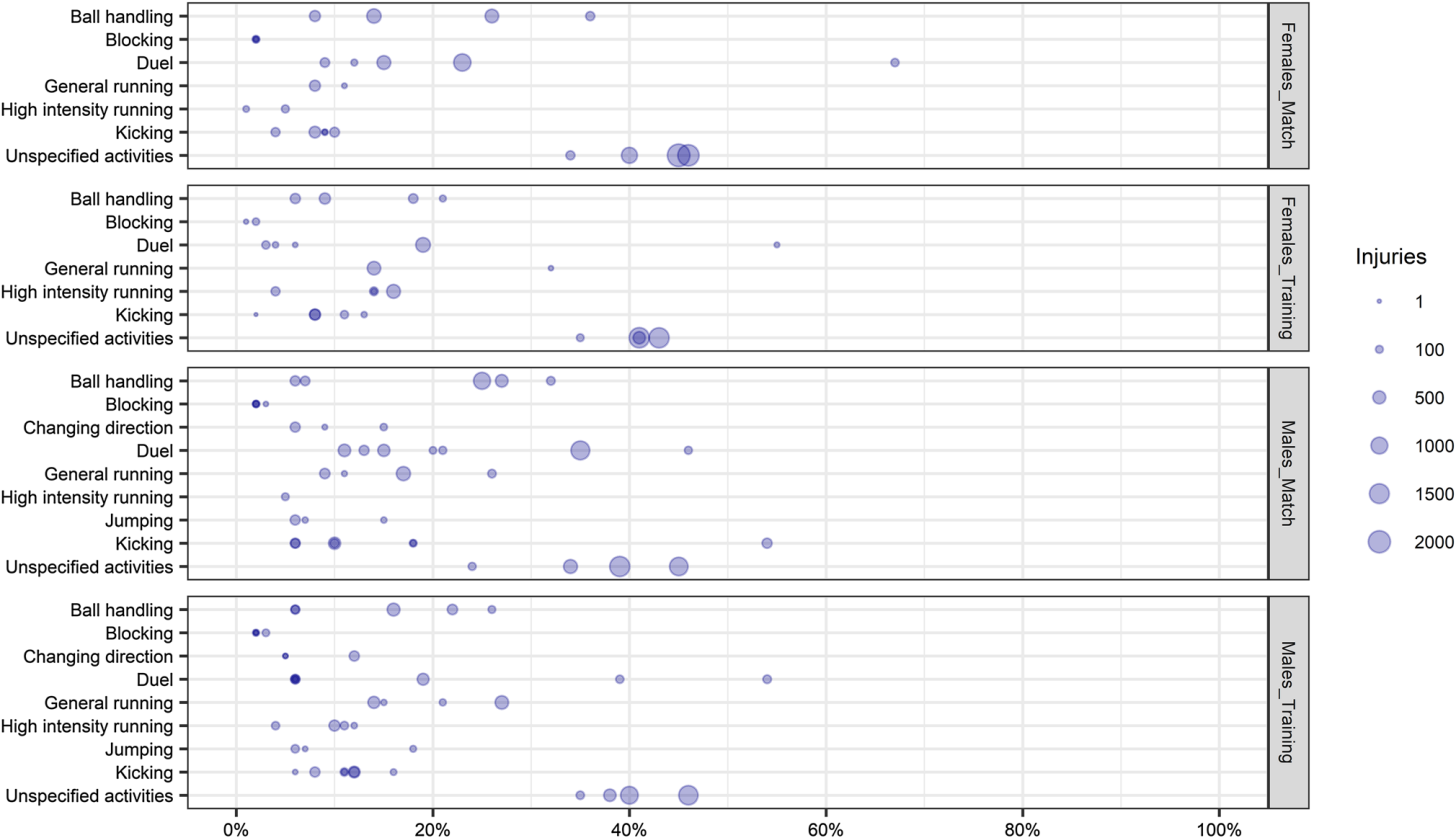
Percentage of injuries occurred during specific inciting activities by sex and session type reported separately for females and males, with division of match and training injuries for both. Size of the dot represents the amount of injuries. Number of injuries reported: Females-match = 11,457, Females-﻿training = 8937, Males-match = 13,589, Males-training = 9979

In males, the categories duels, general running, and unspecified activities were the most prevalent (Fig. 2), and more injuries occurred while the team was in the attacking phase (Fig. 3). Considering pitch positions, results were unclear as the studies implemented different pitch partitions (Table S6, see ESM). Ten studies described the inciting activities according to session type. In males, the proportion of ball handling injuries was slightly higher in matches than in training, while the proportion of unspecified activities and high-intensity running injuries was slightly higher in training than in matches (Fig. 4).

In females, the categories duels and unspecified activities were the most prevalent, while information about pitch position was unclear, also due to different pitch partitions. The percentage of injuries which occurred during ball handling and duels was slightly higher in matches than during training, while the percentage of injuries occurred during high-intensity running was higher in training than in matches.

Eight studies [26, 28, 30, 60–64] analysed the inciting activities using video analysis. In males, duel and high-intensity running were the most prevalent categories (Fig. S1, see ESM). In females, the category ‘duel’ was the most prevalent, while few injuries occurred during kicking, general running, and changing direction.

### Inciting Activities of Specific Injuries

**Fig. 5:**
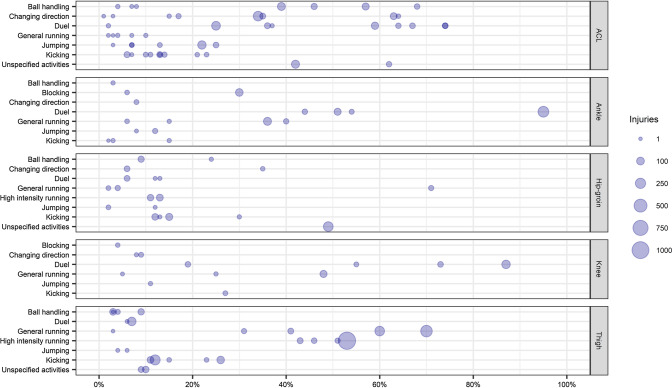
Percentage of injuries occurred during specific inciting activities by injury type. Size of the dot represents the amount of injuries. Knee injuries represent all non-ACL knee injuries. Number of injuries reported: ACL = 1716, Ankle = 593, Hip-groin = 947, Knee (no ACL) = 353, Thigh = 3040

Inciting activities of hip, thigh, knee, and ankle injuries were reported by 29 studies, which allows a specific analysis of the activities of specific injury types. Inciting activities of 1716 ACL injuries were reported by 12 studies. Original data (i.e., as reported by the included studies) are available for the reader in Table 2. A small proportion of injuries occurred during general and high-intensity running, while results for other categories were heterogeneous (Fig. 5). With reference to the inciting activities reported by the studies, changing direction, pressing, tackling, twisting/turning, landing, and being tackled were reported as the most frequent inciting activities of ACL injuries in males (Table 2). Such injuries occurred mainly during the defensive phase but information regarding pitch position was reported inconsistently. In females, tackling, pressing, defending, chasing a loose ball, cutting, and stopping were among the most frequent inciting activities of ACL injuries. Kaneko et al. [33] and Lucarno et al. [43] reported that ACL injuries occurred more frequently during the defensive phase. Five studies [38, 39, 41–43] analysed the inciting activities leading to ACL injuries using video analysis. ‘Duel’ was the most prevalent category, while general running and jumping were the least prevalent (Fig. S2, see ESM). Considering the inciting activities reported by the studies, tackling, recovering the ball, and pressing were reported as the most frequent inciting activities of ACL injuries in males.

Four studies reported inciting activities of 353 other knee injuries with duels, kicking, and general running accounting for the highest proportion of inciting activities. Two studies [48, 55] analysed the inciting activities leading to knee injuries using video analysis. ‘Duel’ was the most prevalent category, while falling, being tackled, pressing, and tackling were the most prevalent inciting activities reported by the studies.

Inciting activities of 3040 hamstring and thigh injuries were reported by six studies. These injuries occurred predominantly during running and kicking activities (Fig. 5). Two studies [59, 60] analysed the inciting activities leading to thigh injuries using video analysis. High-intensity and general running were the most prevalent categories, while sprinting, lunging, and accelerating were the most prevalent inciting activities reported by the studies. Inciting activities of 947 hip/groin and adductor injuries were reported by four studies. Circa 10% of all the injuries occurred during duels, while the percentage was slightly higher for kicking and changes of direction. Results for other inciting activities were inconsistent. Serner et al. [44] analysed the inciting activities of adductor longus injuries using video analysis and reported that such injuries mainly occurred during changing direction or kicking activities in the defensive and midfield thirds. Finally, five studies reported inciting activities of 593 ankle and foot injuries, with duels being the most common (Table 3). Three studies [45, 46, 48] analysed the inciting activities leading to ankle injuries using video analysis. ‘Duel’ was the most prevalent category, while being kicked and contact with another player were the most prevalent inciting activities reported by the studies. The inciting activities reported by all the studies can be explored in detail using the online dashboard available at the following link: https://public.tableau.com/views/SystematicReviewofInjuryIncitingActivitiesinFootball/Story1?:language=en-GB&:display_count=n&:origin=viz_share_link [65].

### Assessment of Risk of Bias

**Fig. 6:**
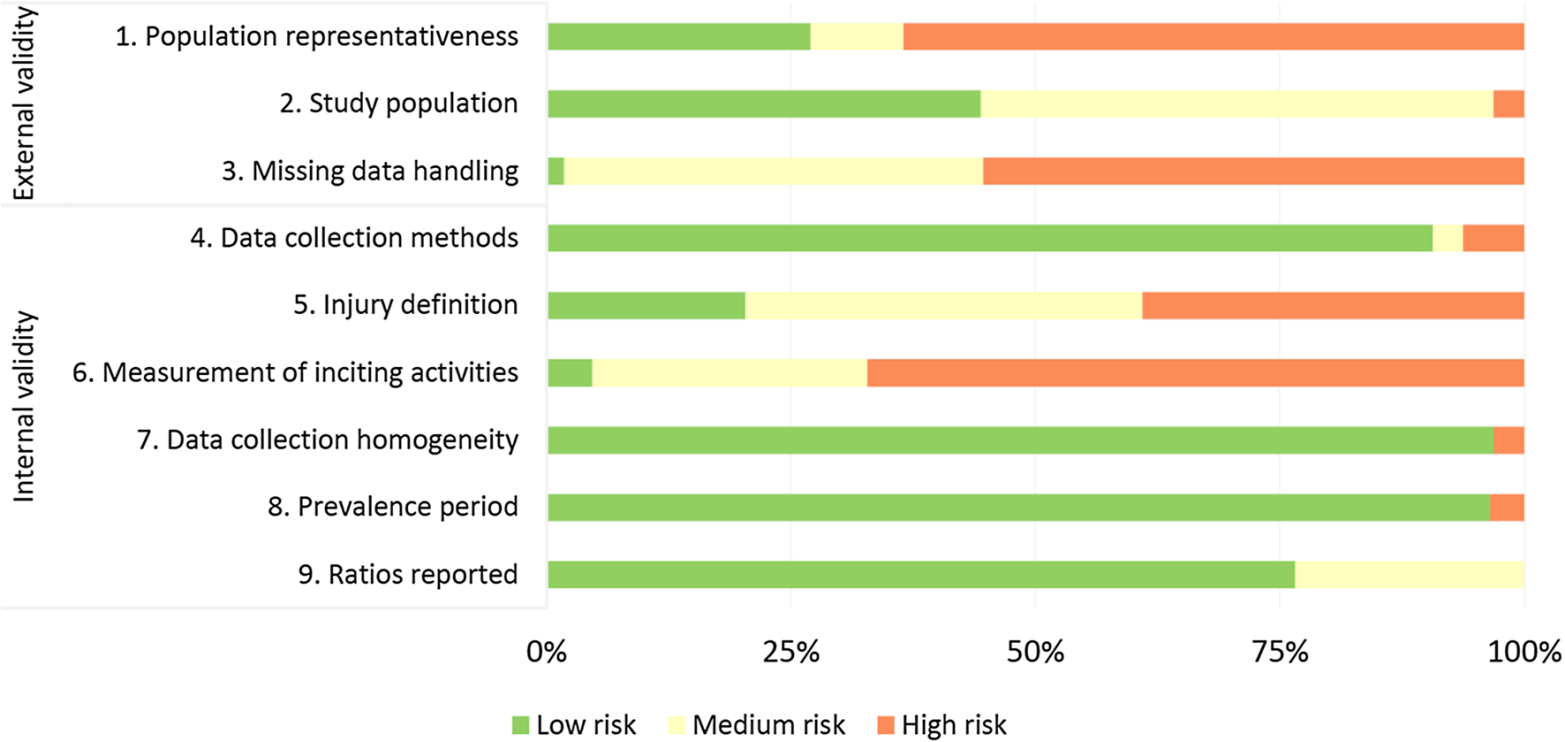
Assessment of risk of bias (RoB) of the included studies

On average, the RoB was deemed as high or medium in 50% of all the items included in the checklist. With respect to external validity, 76% of the items were scored as medium or high RoB and for internal validity, 36% of the items were scored as high RoB (Fig. 6). High RoB was observed in the case definition and in methods for classification and measurement of inciting activities. Only three studies analysed inciting activities with both video analysis and classification using a standardised system, while the remaining studies reported the inciting activities using arbitrary classifications and/or collected these data through interview, questionnaires, or reports without providing further details on how they were collected. Additionally, only 13 studies implemented an appropriate injury definition supported by an appropriate reference, while 13 studies did not report the injury definition at all.

The studies which analysed ACL injuries showed a higher RoB than studies reporting on other injury locations (Fig. S3, see ESM). Specifically, only one study clearly defined the study sample. Furthermore, all the studies that analysed ACL injuries showed high RoB for injury definition and systems implemented to measure inciting activities.

Assessment of risk of bias for each included study is reported in Supplementary File 1 (see ESM).

## Discussion

Due to methodological heterogeneity of the studies, summarising the available literature to provide information about the proportion of inciting activities is challenging. Therefore, the following discussion provides a summary and qualitative comparisons among studies and injury-inciting activities.

### Injury-Inciting Activities in General

Duels and unspecified activities appeared to represent higher risk activities, while blocking and changing direction were reported as inciting activities leading to a small percentage of injuries in both males and females (Fig. 2). It seems that, in the included studies, running activities may have contributed to a higher percentage of injuries in males than in females. Although some differences in inciting activities of injuries between males and females could be expected due to differences in injury characteristics, anthropometric, physiological, and physical performance aspects [66–70], comparisons are currently difficult based on the limited literature available on inciting activities in females’ football. Small differences appear when considering only the studies which analysed the inciting activities through video analysis. Duels clearly represent the riskiest activity and the results for ball handling and blocking activities seem more consistent than those reported by studies which analysed the inciting activities with other methods. However, results with reference to general and high-intensity running remain heterogeneous.

Due to inconsistencies in reporting and to the limited number of studies reporting inciting activities for training and matches separately, data should be interpreted carefully.

Information regarding playing phases in which injuries occurred were reported by eight studies. These studies reported that injuries in males occurred more frequently during the attacking phase than during the defensive phase. It has been observed that players' physical performance changes depending on ball possession [71], which may explain the higher number of injuries occurring during the attacking phase. However, this information has been reported only in a few studies and usually in a generic way, and therefore needs to be further investigated.

### Injury-Inciting Activities for Specific Injury Locations

The inciting activities leading to ACL injuries were analysed by 12 studies (*n* = 6 for male, *n* = 3 for female, *n* = 3 for both), but the results were heterogeneous both in categories used to classify inciting activities and in the original activities reported by the studies. Results appear slightly more consistent when considering only studies which analysed the inciting activities using video analysis. Duels clearly appear as the riskiest activities for ACL injuries followed by kicking. Changing direction, general running, and jumping account for a small percentage of ACL injuries, while data concerning ball handling remain heterogenous. Considering the activities originally reported by the included studies, four studies [40–43] reported pressing as the riskiest inciting activity, followed by regaining balance after kicking, being tackled, and dribbling. The remaining studies which analysed the inciting activities through video analysis [38, 39] did not report any injury occurring during pressing or regaining balance after kicking, but reported ball recovery, tackling, and cutting as the most prevalent inciting activities. It has been largely reported in the literature that the load at which the ACL is exposed to increases when the knee is in valgus, intra-rotated, and extended position [5, 72, 73], which could occur during changes of direction [74] and when players perform a tackle [75]. Therefore, it would be expected to observe that these inciting activities contribute to a high percentage of ACL injuries, but this was not always the case. Indeed, duel activities and changing direction contributed to a high percentage of injuries in some of the included studies, but in others no injuries occurred during these activities. This may be caused by the different classifications used to report the inciting activities. Even if changing direction and tackling are expected to be among the main inciting activities of ACL injuries, the results currently available in males and females cannot confirm this hypothesis, and it therefore needs to be investigated further.

The inciting activities of thigh injuries were reported by four studies, and two studies specifically reported the inciting activities leading to hamstring injuries. Running activities were the most reported inciting activities of these injuries, accounting for more than half of the total number of injuries. Kicking activities seem to be the second main inciting activities of thigh and hamstring injuries. This partially aligns with the results reported by two studies that used video analysis to evaluate the inciting activities of thigh [60] and specifically hamstring injuries [59], reporting that general and high-intensity running are the most prevalent activities leading to thigh injuries. Of the activities originally reported by the included studies, sprinting and running were the most prevalent activities leading to thigh injuries while lunging and accelerating were the most prevalent activities leading to hamstring injuries. These results are partially in accordance with what has previously been hypothesised for football injuries and reported in other sports for hamstring [76–78] and rectus femoris injuries [79]. Running activities are believed to be the main cause of hamstring injuries followed by kicking activities [80]. However, Gronwald et al. [59] reported that lunging was the most prevalent activity leading to hamstring injury followed by accelerating, high-speed running, and kicking. For rectus femoris injuries, it has been hypothesised that kicking and high-intensity activities such as accelerating, decelerating, and running at high speed may put the rectus femoris at risk of injury [79]. This is partly supported by the results reported by Klein et al. [60], which suggested that sprinting, running, and lunging are the most prevalent activities leading to thigh injuries, although the investigators did not report the specific injury location (e.g., rectus femoris, hamstring).

Even if the results of this review seem to confirm what has been hypothesised in football and reported in other sports, they should be interpreted carefully given several limitations of the included studies. While Cross et al. [49] and Gronwald et al. [59] clearly defined the injuries analysed (i.e., hamstring), the remaining studies reported inciting activities of thigh injuries in general, which could include different muscle groups (e.g., hamstring, quadriceps), and therefore it is not clear which injuries occurred during the reported inciting activities. Furthermore, the number of studies which analysed such injuries is limited and they only reported generic descriptions of their inciting activities (e.g., running, kicking), which provide limited information. For example, it would be useful to analyse the running phase and the running speed at which injuries occur. In runners, it has been reported that hamstring injuries occur while the athletes are running near their maximal speed [82], but such information has been reported by only four studies here [50, 58–60], reporting high-intensity running without specifying the running speed. Furthermore, there is debate on whether running injuries occur during the early stance or the swing phase of running [80, 83]. Similarly, generic information was also reported for injuries which occurred during kicking activities. Indeed, only the activities performed were evidenced (e.g., shooting, passing), but no details were presented on the kicking phase in which injuries occurred. These details may be important to achieve a more complete understanding of the injury-inciting activities because, as reported by Mendiguchia et al. [79], rectus femoris injuries may occur during one of the three phases of kicking (i.e., swing phase, ball contact phase, ground contact phase). Providing such detailed information would be helpful for football practitioners, who could use them to plan training sessions and to develop injury prevention strategies [84], although we recognise that gathering this information could be difficult unless it is provided by the injured players themselves.

Inciting activities of hip/groin injuries were described by four studies. In the studies included in this review, high-intensity running, kicking, and duel activities were reported as the inciting activities for 15% of hip/groin injuries each, while results of the other inciting activities (e.g., changing direction, general running) were unclear. Serner et al. [44] analysed the activities leading to adductor longus injuries through video analysis and reported that changing direction and kicking were the most prevalent activities leading to injury. These results are partially consistent with the available literature on the biomechanics of adductor injuries. Indeed, the adductor longus, reported as having a higher incidence compared with the other adductor muscles [44, 85], achieves its peak eccentric activation when the hip is close to its maximal extension [86], thought to put the adductor longus at high risk of injury [81, 86]. Considering that this position is achieved during kicking and running activities [86, 87], they are expected to be the most common inciting activities of the adductors injuries.

In addition to kicking and running at high intensity, it is believed that changing direction may be another injury activity of adductor injuries. This type of injury seems to be more common in sports involving accelerations, decelerations, and changes of direction [88, 89], and it is thought to be linked to this muscle group experiencing high eccentric load when the leg is abducted and externally rotated [85], as seen during execution of changes of direction [90]. However, the studies included in the current review reported inconsistent results regarding this activity. Serner et al. [44] reported that 35% of adductor injuries occurred during changing direction, but this was the reported inciting activity in only 6% of the injuries in the study conducted by Lundgårdh et al. [51] and was not reported for any other football study involving hip/groin injuries [52, 56]. These differences could be due to different injury types analysed by the included studies; however further investigations are needed.

Regarding other specific injury locations, five studies reported the inciting activities of ankle and foot injuries; however, the reported inciting activities were mainly inconsistent, although the risk of incurring this type of injury seems to be slightly higher in duel activities. Three studies analysed ankle injuries using video analysis and they all reported duel activities (i.e., contact with another player, being tackled, and tackling) as the activities leading to more than half of the injuries analysed [45, 46, 48]. Kofotolis et al. [45] and Andersen et al. [46] reported that around 8% of injuries occurred during landing but Krutsch et al. [48] did not report any injury occurring during landing. These results seem to be in contrast with the literature available for ankle injuries in other sports. Within a systematic review, it has been reported that these injuries occur mainly during non-contact activities [91]. Specifically, studies suggest that ankle sprains, which constitute the majority of ankle injuries, occur with the foot in plantar flexion commonly occurring during activities such as landing and changing direction [92, 93]. In basketball, such injuries seem to occur mainly during landing (45%) and changing direction (30%), while only 10% of these injuries occur in contact activities [94].

### Methodological Considerations on the Selected Studies and Recommendations for Future Research

Several methodological limitations were observed in the studies included in this review, 76% and 36% of the items evaluating external and internal validity, respectively, were scored as medium or high RoB. The external validity was mainly influenced by the fact that 73% of the studies did not clearly and explicitly specify the target population (i.e., population to which the researchers would like to apply the results) and hence it is difficult to understand whether the study population was a close representation of the target population (or acceptable for scientific inference and hence for generalisation). Fifty-five percent of studies did not clearly report information such as age, country of competition, competitive level, and number of teams and participants included in the study. This makes it difficult to understand which population the results could be applied to. For example, it has been reported that physical performance changes according to age [95–97] and competitive level [98], and therefore the proportion of the inciting activities may change according to such player characteristics.

The internal validity of the studies was mainly influenced by methods for collecting and reporting injury data. Firstly, 45% of the included studies which analysed ACL injuries, 24% of studies which analysed other specific injuries, and 11% of studies which analysed general injuries collected data from databases or online platforms and had limited or no access to reliable medical information being directly provided by the medical staff, which, except for severe injuries (e.g., ACL) for which this method seems reliable, may limit the validity of the results [12, 99, 100]. This approach is being increasingly used, but we suggest consideration of its limitations before doing so, because some injuries may not be captured on video and online information may not be reliable. Krosshaug et al. [12] reported that studies which analysed injuries through video analysis only may have missed up to 70% of the non-contact injuries and up to half of the total injuries that occurred. This may happen because some injuries, especially non-contact injuries, may occur far from the ball and are therefore not being captured by the footage, or because players keep playing for some time before reporting the injury to the medical staff and therefore locating the inciting activity is difficult. Furthermore, Krutsch et al. [99] suggested that non-severe injury data reported online have low validity and recommended using these data only after verification.

Secondly, most of the studies included in the review did not implement an appropriate definition of injury and/or a validated or standardised classification system for the inciting activities. As reported by other systematic reviews [101–105], many studies analysing football injuries do not follow the guidelines on injury definition provided by consensus statements such as Fuller et al. [31]. These guidelines are not necessarily the best way to classify injuries, but the heterogeneity of injury classification could substantially influence the total number of injuries and therefore the number of injuries occurring during each inciting activity. For example, the number of injuries occurring during activities that lead mainly to minimal and mild injuries (i.e., lasting < 8 days) may change significantly according to the injury definition implemented, as these injuries may not be considered in studies which consider players injured only if they miss a match [106]. However, the main limitation observed in the included studies was the use of non-standardised systems to classify the inciting activities. This led the researchers to classify and report such data using arbitrary classifications, which is among the main causes of the heterogeneous results making the comparisons difficult between studies. Indeed, more than 100 different inciting activities have been reported, with some being used only by a few studies. Additionally, some of the inciting activities reported, such as general play and contact with another player, are quite generic and provide limited information. To reduce such heterogeneity and compare the results of the studies, it was necessary to (arbitrarily) group inciting activities into categories, which limited the level of detail of the analysis.

Thirdly, most of the included studies did not specify whether the injured players were performing more than one activity when the injuries occurred. For example, kicking injuries can occur with the players running at high speed (e.g., player kicking the ball with the first touch after a long pass) or in a more static situation (e.g., penalty or corner kick). It is unclear how injuries that occurred in mixed activities (e.g., kicking while running at high speed) were reported; however, it would be appropriate to report all the activities performed at the time of injury as the combination of different activities may help to better understand why the injury occurred.

Finally, most of the studies did not specify for each inciting activity how many injuries occurred during contact and non-contact circumstances, which is another important limitation. The few studies that reported such information did not identify the nature of the contact (i.e., direct or indirect contact). These limitations could have led to a misclassification of the injuries and could have influenced the analysis of proportion [107].

Future research on inciting activities should try to avoid the limitations mentioned above. First of all, it is paramount that studies implement standardised injury definitions and standardised systems to classify the inciting activities in order to have consistent data that can be compared among studies. Fuller et al. [31] provided guidelines for injury definitions to implement in football. To the best of our knowledge, the only standardised system to classify inciting activities currently available is the football incident analysis developed by Andersen et al. [62]. However, this method has been developed specifically for video analysis and may be time demanding, which could be a barrier to its implementation, particularly in the practical setting of football teams. Indeed, to our knowledge it has been implemented by only three studies since its development in 2003 [28, 30, 62]. Therefore, it may be appropriate to consider the development of alternative methods for the classification of inciting circumstances in football using report forms and video analysis, which despite some limitations are considered the most practical approaches for the analysis of inciting circumstances [12].

Additionally, reporting details on the session in which the injury occurred and on injury type would allow more detailed analyses to be performed. Specifically, reporting the inciting activities according to the injury type (e.g., ACL, hamstring, ankle) could allow the readers to understand the activities that can lead to specific injuries. Furthermore, specifying whether the injuries occurred during training or matches could allow a comparison among inciting activities during these two types of session and could inform on the activities and circumstances which need to be limited or further trained to reduce the risk of injury. Finally, also including details on the nature of contact (i.e., direct contact, indirect contact, non-contact) as suggested by existing reporting guidelines [31, 107], which is sometimes referred to as *injury mechanism,* would allow even more detailed analyses.

### Limitations

Despite following the PRISMA 2020 guidelines as well as addressing the AMSTAR 2 domains, this review is not exempt from limitations. Although a comprehensive research strategy was implemented, it is possible that some studies which reported inciting activities were not identified in this systematic review. This may be due to the fact that such information is usually reported in the full text as additional analysis, without being cited in the title or abstract. The exclusion of studies that were not written in English might be an additional cause. The inclusion of studies that analysed inciting activities without this being their main aim (i.e., as additional analysis) might be seen as a limitation. However, we believe that practitioners and researchers also use additional analyses from scientific studies to inform their practice, and we therefore deemed it necessary to include such studies in the present review. Nevertheless, not being the main outcome, the accuracy of these results may be inferior and this should be considered when interpreting the results. Despite our attempt to group similar inciting activities, this categorisation remains arbitrary, and their interpretation could have been affected by the heterogeneity of the methods implemented by the included studies. The lack of standardised methods and of studies on specific injuries did not allow us to perform a meta-analysis. For the same reasons, it was not possible to analyse the results according to age and playing level. Finally, the tool to assess the RoB in the studies had to be adapted from existing instruments, as there were none available that allowed us to address the domains deemed relevant. Therefore, the cut-off values between low, medium, and high risk of bias are not validated, and thus come with some uncertainty.

## Conclusions

Duels and unspecified activities accounted for the highest proportion of injury-inciting activities in general. High-intensity running and kicking activities might be the main inciting activities of thigh and groin injuries, while duels may be the most common inciting activities of ankle injuries. Duel activities and pressing may be the most common inciting activities leading to ACL injuries, but there is not complete agreement within the literature.

These results need to be interpreted carefully, as the available evidence is limited and as most of the included studies implemented non-validated methods for the collection of injury data and for the analysis of the inciting activities, which led to heterogeneous results among studies. In turn, recommendations for injury prevention strategies based on inciting events are difficult.

Further studies should collect and analyse injury data using standardised methods and should report more details on the injuries and the activities and circumstances leading to injury. This would increase the consistency of data and allow a comparison between studies and help the understanding of inciting activities within football. It seems necessary to develop a classification system to allow practitioners and researchers to systematically report and analyse injury-inciting activities in football.

## Supplementary Information

Below is the link to the electronic supplementary material.

Supplementary file1 (DOCX 733 kb)

Supplementary file2 (XLSX 12 kb)

## Declarations

### Availability of data and material

All data generated or analysed during this study are included in this published article (and its supplementary information files).
