# Supplementary material for: Injury-Inciting Activities in Male and Female Football Players: A Systematic Review
Source: Sports Med. 2022 Oct 31;53(1):151–76. doi: 10.1007/s40279-022-01753-5 (PMC9807506; doi:10.1007/s40279-022-01753-5)
Supplement: Supplementary file 1 — Supplementary file1 (DOCX 733 kb) [file 40279_2022_1753_MOESM1_ESM.docx]

# Appendices

Table S1 Search strategy for each database

| **Database** | **Search Strategy** | **Results (n)** |
| --- | --- | --- |
| PubMed | (((injur*) AND (((((((mechanism*) OR event*) OR situation*) OR circumstance*) OR occasion*) OR activit*) OR characteristic*)) AND ((((competition*) OR game*) OR match*) OR training)) AND ((football) OR soccer) | 1872 |
| SPORTDiscus | "ALL FIELDS: (injur*) AND ALL FIELDS: (((((((mechanism*) OR event*) OR situation*) OR circumstance*) OR occasion*) OR activit*) OR characteristic*) AND ALL FIELDS: ((football) OR soccer) AND ALL FIELDS: ((((competition*) OR game*) OR match*) OR training)  Refined by: LANGUAGES: ( ENGLISH ) AND DOCUMENT TYPES: ( ARTICLE )" | 1654 |
| Web of Science | #1  ALL FIELDS: (training) OR ALL FIELDS:(match*) OR ALL FIELDS: (game*) OR ALL FIELDS: (competition*)  Indexes=SCI-EXPANDED, SSCI, A&HCI, CPCI-S, CPCI-SSH, BKCI-S, BKCI-SSH, ESCI, CCR-EXPANDED, IC Timespan=All years  #2  ALL FIELDS: (injur*)  #3  ALL FIELDS: (mechanism*) OR ALL FIELDS: (event*) OR ALL FIELDS: (circumstance*) OR ALL FIELDS: (occasion*) OR ALL FIELDS: (activit*) OR ALL FIELDS: (situation*) OR ALL FIELDS: (characteristic*)  Indexes=SCI-EXPANDED, SSCI, A&HCI, CPCI-S, CPCI-SSH, BKCI-S, BKCI-SSH, ESCI, CCR-EXPANDED, IC Timespan=All years  #4  ALL FIELDS: (football) OR ALL FIELDS: (soccer)  Indexes=SCI-EXPANDED, SSCI, A&HCI, CPCI-S, CPCI-SSH, BKCI-S, BKCI-SSH, ESCI, CCR-EXPANDED, IC Timespan=All years  #5  #4 AND #3 AND #2 AND #1  Refined by: LANGUAGES: ( ENGLISH ) AND DOCUMENT TYPES: ( ARTICLE ) AND DOCUMENT TYPES: ( ARTICLE )  Indexes=SCI-EXPANDED, SSCI, A&HCI, CPCI-S, CPCI-SSH, BKCI-S, BKCI-SSH, ESCI, CCR-EXPANDED, IC Timespan=All years | 2203 |
| OpenGrey | (((injur*) AND (((((((mechanism*) OR event*) OR situation*) OR circumstance*) OR occasion*) OR activit*) OR characteristic*)) AND ((((competition*) OR game*) OR match*) OR training)) AND ((football) OR soccer) | 5 |

Table S2 Tool to assess Risk of Bias. Adapted from Hoy et al. [21] and National Institutes of Health [22]

| **N.** | **Item description** | **Criteria** | **How the item can influence the results of the review** | **Examples** |
| --- | --- | --- | --- | --- |
| External validity | | | | |
| 1 | Was the study’s target population a close representation of the national target in relation to relevant variables, e.g. age, sex, occupation? | Study participants should be of same or similar nationality, age, and competitive level of the target population | If study population was not a close representation of target population results of the study may not be applicable to the target population | • In South Africa, there are few studies on high school female soccer players injuries. 200 female high school players competing from 6 different schools were included in the study: Yes (Low Risk of Bias)  • There is little information on mechanisms of injury in European professional football. 200 amateur players competing in 4 different countries were included in the study: Partial (Medium Risk of Bias)  • There is little information on mechanisms of injury in European professional football.. 200 amateur players competing in English football clubs were included in the study: No (High Risk of Bias)  • There is little information on mechanisms of injury in football. 200 amateur players competing in English football clubs were included in the study: Unclear (High Risk of Bias) |
| 2 | Was the study population clearly specified and defined? | Number of teams and participants, age, sex, country of competition and competitive level of the population from which study participants were recruited should be stated. | If study population is not well defined, it may be hard understanding the external validity of the findings. | • Data were collected from 100 male amateur football players (age 18 - 32) competing in 4 teams of the 5th Italian division: Yes (Low Risk of Bias)   • Data were collected from 100 football players (age 18 - 32) competing in the 5th Italian division: Partially (Medium Risk of Bias)   • Data were collected from 100 football players: No (High Risk of Bias) |
| 3 | Were missing data addressed with appropriate methods? | If there are missing data, it should be reported the number of missing data and whether there were significative differences among cases and participants with and without missing data | Missing data can bias the prevalence of mechanisms of injury | • Information on mechanisms of injury were missing for 5 cases (2%). However the researchers found no significative differences between players and injury data with and without missing data: Yes (Low Risk of Bias)  • Information on mechanisms of injury were missing for 100 cases (40%): Partially (Medium Risk of Bias)  • The cases with missing data for mechanisms of injury were excluded from the analysis: No (High Risk of Bias)  • No information are reported for missing data: Unclear (High Risk of Bias)  • Injury data were searched by internet search: NA |

| Internal validity | | | | |
| --- | --- | --- | --- | --- |
| 4 | Were data collected directly from the participants (as opposed to a proxy)? | Injury data must directly come from players or staff (i.e., not coming from databases or internet sources) or from online databases in cases of severe injuries | Data coming from databases or internet sources may come from untrustworthy sources and have been reported or collected with various and inappropriate methods. This may afflict data reliability and therefore study results. | • Data were collected by team doctor during team training and matches: Yes (Low Risk of Bias)   • Data on severe injuries were collected from online databases (e.g., Transfermarkt): Yes (Low Risk of Bias)  • Data on non-severe injuries were collected from online databases (e.g., Transfermarkt): No (High Risk of Bias) |
| 5 | Was an acceptable case definition used in the study? | Injury definition must be acceptable, clearly stated and justified by a validation reference (e.g. Fuller 2006). | An unclear or inappropriate case definition may influence the number of cases and therefore the prevalence of each mechanism | • Following the guidelines reported by Fuller et al., (2006), injuries were defined as any physical complaint sustained  by a player that resulted from a football game or training session and led to them being unable to take a full part in future football training or match play: Yes (Low Risk of Bias)  • As previously done in other studies, injuries were defined as any physical complaint sustained  by a player that resulted from a football game or training session: Partially (Medium Risk of Bias)  • Injuries were defined as receiving medical treatment on the pitch: N (High Risk of Bias)  • Injury definition is not reported: U (High Risk of Bias) |
| 6 | Was the study instrument that measured the parameter of interest shown to have validity and reliability? | Activities of injury must have been analysed through video analysis and classified using a standardised system (e.g., FIA) | The usage of non-validated methods to evaluate injury activities may limit validity of the results and afflict prevalence of mechanisms | • Injury activities were evaluated through video-analysis and classified using a standardised classification system: Yes (Low Risk of Bias)  • Injury activities were evaluated through video-analysis: Partially (Medium Risk of Bias)  • Injury activities were evaluated through report/questionnaires: No (High Risk of Bias) |

| 7 | Was the same mode of data collection used for all participants? | Data related to injuries must have been collected through the same mode for all the participants | The usage of different methods to collect injury data may alter injury prevalence | • Injury activities were evaluated through report only: Yes (Low Risk of Bias)  • Injury activities were evaluated through video-analysis and report. Data obtained through each method are reported separately in table: Partially (Medium Risk of Bias)  • Injury activities were evaluated through video-analysis and, when video clip were not available, data were obtained by medical reports: No (High Risk of Bias)  • Injury activities were evaluated through report and video-analysis: Unclear (High Risk of Bias) |
| --- | --- | --- | --- | --- |
| 8 | Was the length of the shortest prevalence period for the parameter of interest appropriate? | At least 1 entire season or 1 entire tournament should have been analysed | An inappropriate length of prevalence/observation period may bias injury prevalence | • Data were collected during 2 seasons: Yes (Low Risk of Bias)   • Data were collected during 10 matches: No (High Risk of Bias)   • The study does not indicate the length of observation period: Unclear (High Risk of Bias) |
| 9 | Were the numerator(s) and denominator(s) for the parameter of interest appropriate? | Appropriate numerator (number of injuries for a given mechanism) and denominator (total number of injuries) should have been reported. If percentages are reported without reporting the numerator, score as "unclear". | An unclear reporting of numerators and denominators may limit the understanding of prevalence | • In total, 100 injuries occurred. 20 injuries occurred while running, 10 occurred while kicking, etc: Yes (Low Risk of Bias)   • In total, 100 injuries occurred. 20 % of total injuries occurred while running, 10 % occurred while kicking, etc: Partially (Medium Risk of Bias)   • The study does not indicate the length of observation period: Unclear (High Risk of Bias) |

Table S3 **I**nter-rater agreement in the analysis of RoB

| Round | Number of studies | Studies | Percentage of agreement | Cohen’s Kappa |
| --- | --- | --- | --- | --- |
| Familiarisation | 2 | [38, 108] | NA | NA |
| 1 | 8 | [26, 29, 40, 41, 63, 64, 109, 110] | 88% | 0.81 |
| 2 | 5 | [28, 35, 111-113] | 87% | 0.67 |
| 3 | 5 | [45, 57, 61, 114, 115] | 91% | 0.87 |
| 4 | 8 | [27, 34, 52, 54, 58, 116-118] | 92% | 0.87 |
| 5 | 5 | [43, 48, 50, 60, 119] | 98% | 0.92 |

Table S4

List of full texts excluded with reason (n = 142)

| **Title** | **Authors** | **Year** | **Reason of exclusion** | |
| --- | --- | --- | --- | --- |
| A 15-year prospective epidemiological account of acute traumatic injuries during official professional soccer league matches in Japan | H. Aoki; N. O'Hata; T. Kohno; T. Morikawa; J. Seki | 2012 | Inciting activities not reported | |
| A comparative analysis of injuries in handball, hockey, volleyball and soccer in kenya | M. Wekesa; W. W. Njororai; E. L. Madaga; J. M. Asembo | 2001 | Unable to access | |
| A Decade of Hip Injuries in National Collegiate Athletic Association Football Players: An Epidemiologic Study Using National Collegiate Athletic Association Surveillance Data | J. L. Makovicka; A. Chhabra; K. A. Patel; S. V. Tummala; D. E. Hartigan | 2019 | Other sport | |
| A four year prospective study of injuries in elite Ontario youth provincial and national soccer players during training and matchplay | M. Mohib; N. Moser; R. Kim; M. Thillai; R. Gringmuth | 2014 | Inciting activities not reported | |
| A one year prospective study of soccer injuries in the 1992-1993 Kenyan national team | M. Wekesa | 1995 | Unable to access | |
| Analysis of injury incidences in the Korea national men's soccer teams | K. Hwang-Bo; C. H. Joo | 2019 | Inciting activities not reported | |
| Analysis of injury incidences in the Korea national men's soccer teams | K. Hwang-Bo; C. H. Joo | 2019 | Inciting activities not reported | |
| Ankle injuries among United States high school sports athletes, 2005-2006 | A. J. Nelson; C. L. Collins; E. E. Yard; S. K. Fields; R. D. Comstock | 2007 | Mixed population | |
| Ankle injuries in football academies: a three-centre prospective study | D. J. Cloke; P. Ansell; P. Avery; D. Deehan | 2011 | Inciting activities not reported | |
| Ankle sprain in athletes: retrospective study | M. F. Pedrini; F. Petraglia; O. Licari; C. Costantino | 2013 | Inciting activities not reported | |
| Anterior cruciate ligament injury in elite football: a prospective three-cohort study | M. Walden; M. Hagglund; H. Magnusson; J. Ekstrand | 2011 | Inciting activities not reported | |
| Anterior cruciate ligament injury in female athletes: Epidemiology | M. L. Ireland | 1999 | Review | |
| Anterior cruciate ligament injury patterns and their relationship to fatigue and physical fitness levels - a cross-sectional study | S. F. Alsubaie; W. K. Abdelbasset; A. A. Alkathiry; W. M. Alshehri; M. M. Azyabi; B. B. Alanazi; A. A. Alomereni; F. Y. Asiri | 2021 | Population |  |
| Aspects on musculo-skeletal trauma incidence in competitive sportsmen. A comparative study of athletes and football players - part ii | M. Elena-Doina; M. Alexandra | 2012 | Other sport | |
| Association between ball-handling versus defending actions and acute noncontact lower extremity injuries in high school basketball and soccer | S. M. Monfort; R. D. Comstock; C. L. Collins; J. A. Onate; T. M. Best; A. M. Chaudhari | 2015 | Inciting activities not reported | |
| Avoidance of soccer injuries with preseason conditioning | R. S. Heidt, Jr.; L. M. Sweeterman; R. L. Carlonas; J. A. Traub; F. X. Tekulve | 2000 | Inciting activities not reported | |
| Boys soccer league injuries: a community-based study of time-loss from sports participation and long-term sequelae | T. Timpka; O. Risto; M. Bjormsjo | 2008 | Inciting activities not reported | |
| Changes in injury incidences and causes in Swiss amateur soccer between the years 2004 and 2015 | A. Gebert; M. Gerber; U. Puhse; O. Faude; H. Stamm; M. Lamprecht | 2018 | Inciting activities not reported | |
| Comparative sport injury epidemiological study on a Spanish sample of 25 different sports | C. Pujals; V. J. Rubio; M. O. Marquez; I. Sanchez; R. Ruiz-Barquin | 2016 | Inciting activities not reported | |
| Descriptive epidemiology of collegiate men's soccer injuries: National Collegiate Athletic Association Injury Surveillance System, 1988-1989 through 2002-2003 | J. Agel; T. A. Evans; R. Dick; M. Putukian; S. W. Marshall | 2007 | Inciting activities not reported | |
| Descriptive epidemiology of collegiate women's soccer injuries: National Collegiate Athletic Association Injury Surveillance System, 1988-1989 through 2002-2003 | R. Dick; M. Putukian; J. Agel; T. A. Evans; S. W. Marshall | 2007 | Inciting activities not reported | |
| Descriptive Epidemiology of Soccer Injury During Elite International Competition in Africa | A. Nuhu; M. Kutz | 2017 | Inciting activities not reported | |
| English Premiership Academy knee injuries: Lessons from a 5 year study | O. Moore; D. Cloke; P. Avery; I. Beasley; D. Deehan | 2011 | Inciting activities not reported | |
| Epidemiologic study of young soccer player's injuries in U12 to U20 | C. Tourny; S. Sangnier; T. Cotte; R. Langlois; J. Coquart | 2014 | Inciting activities not reported | |
| Epidemiological Findings of Soccer Injuries During the 2017 Gold Cup | J. Chahla; B. Sherman; M. Cinque; A. Miranda; W. E. Garrett; G. Chiampas; H. O'Malley; M. B. Gerhardt; B. R. Mandelbaum | 2018 | Inciting activities not reported | |
| Epidemiological Patterns of Ankle Sprains in Youth, High School, and College Football | D. R. Clifton; R. M. Koldenhoven; J. Hertel; J. A. Onate; T. P. Dompier; Z. Y. Kerr | 2017 | Other sport | |
| Epidemiological profile of sports-related knee injuries in northern India: An observational study at a tertiary care centre | R. John; M. S. Dhillon; K. Syam; S. Prabhakar; P. Behera; H. Singh | 2016 | Inciting activities not reported | |
| Epidemiology of 10,000 high school football injuries: patterns of injury by position played | M. A. Badgeley; N. M. McIlvain; E. E. Yard; S. K. Fields; R. D. Comstock | 2013 | Other sport | |
| Epidemiology of anterior cruciate ligament injuries in soccer | J. M. Bjordal; F. Arnly; B. Hannestad; T. Strand | 1997 | Inciting activities not reported | |
| Epidemiology of Anterior Cruciate Ligament Injury in Italian First Division Soccer Players | A. Grassi; L. Macchiarola; M. Filippini; G. A. Lucidi; F. Della Villa; S. Zaffagnini | 2019 | Inciting activities not reported | |
| Epidemiology of Anterior Cruciate Ligament Injury in Italian First Division Soccer Players | A. Grassi; L. Macchiarola; M. Filippini; G. A. Lucidi; F. Della Villa; S. Zaffagnini | 2020 | Inciting activities not reported | |
| Epidemiology of football injuries in Asia: a prospective study in Qatar | C. Eirale; A. Farooq; F. A. Smiley; J. L. Tol; H. Chalabi | 2013 | Inciting activities not reported | |
| Epidemiology of Football Injuries in the National Collegiate Athletic Association, 2004-2005 to 2008-2009 | Z. Y. Kerr; J. E. Simon; D. R. Grooms; K. G. Roos; R. P. Cohen; T. P. Dompier | 2016 | Other sport | |
| Epidemiology of Hip and Groin Injuries in Collegiate Athletes in the United States | Y. E. Kerbel; C. M. Smith; J. P. Prodromo; M. I. Nzeogu; M. K. Mulcahey | 2018 | Inciting activities not reported | |
| Epidemiology of injuries in First Division Spanish football | J. Noya Salces; P. M. Gómez-Carmona; L. Gracia-Marco; D. Moliner-Urdiales; M. Sillero-Quintana | 2014 | Inciting activities not reported | |
| Epidemiology of Injuries in First Division Spanish Women's Soccer Players | R. Martín-San Agustín; F. Medina-Mirapeix; A. Esteban-Catalán; A. Escriche-Escuder; M. Sánchez-Barbadora; J. C. Benítez-Martínez | 2021 | Inciting activities not reported |  |
| Epidemiology of injury in English Professional Football players: A cohort study | A. Jones; G. Jones; N. Greig; P. Bower; J. Brown; K. Hind; P. Francis | 2019 | Inciting activities not reported | |
| Epidemiology of meniscal injuries in US high school athletes between 2007 and 2013 | J. Mitchell; W. Graham; T. Best; C. Collins; D. Currie; R. Comstock; D. Flanigan; T. M. Best; D. W. Currie; R. D. Comstock; D. C. Flanigan | 2016 | Inciting activities not reported | |
| Epidemiology of Muscle Injuries in Professional Football (Soccer) | J. Ekstrand; M. Hägglund; M. Waldén | 2011 | Inciting activities not reported | |
| Epidemiology of physical activity-related injuries in Chinese university students | W. Cai; L. Gao; L. Li; Y. Gao; C. Jia; W. Yang; S. Duan; H. Zhang | 2019 | Inciting activities not reported | |
| Epidemiology of player--player contact injuries among US high school athletes, 2005-2009 | Z. Y. Kerr; C. L. Collins; S. K. Fields; R. D. Comstock | 2011 | Inciting activities not reported | |
| Epidemiology of Quadriceps Strains in National Collegiate Athletic Association Athletes, 2009-2010 Through 2014-2015 | T. G. Eckard; Z. Y. Kerr; D. A. Padua; A. Djoko; T. P. Dompier | 2017 | Inciting activities not reported | |
| Epidemiology of soccer players traumatic injuries during the 2015 America Cup | O. Pangrazio; F. Forriol | 2016 | Inciting activities not reported | |
| Epidemiology of time loss groin injuries in a men's professional football league: a 2-year prospective study of 17 clubs and 606 players | A. B. Mosler; A. Weir; C. Eirale; A. Farooq; K. Thorborg; R. J. Whiteley; P. Hlmich; K. M. Crossley | 2018 | Inciting activities not reported | |
| Evaluating the level of injury in English professional football using a risk based assessment process | S. Drawer; C. W. Fuller | 2002 | Data used in another study included | |
| Evaluation of Muscle Injuries in Professional Football Players: Does Coach Replacement Affect the Injury Rate? | G. Donmez; S. Kudas; M. Yorubulut; M. Yildirim; N. Babayeva; S. S. Torgutalp | 2018 | Inciting activities not reported | |
| Exposure to injury in major college football. A preliminary report of data collection to determine injury exposure rates and activity risk factors | B. R. Cahill; E. H. Griffith | 1979 | Other sport | |
| Football injuries at Asian tournaments | Y. S. Yoon; M. Chai; D. W. Shin | 2004 | Inciting activities not reported | |
| Football injuries during European Championships 2004-2005 | M. Walden; M. Hagglund; J. Ekstrand | 2007 | Inciting activities not reported | |
| Football injuries during the 2014 FIFA World Cup | A. Junge; J. Dvorak | 2015 | Inciting activities not reported | |
| Football injuries during the World Cup 2002 | A. Junge; J. Dvorak; T. Graf-Baumann | 2004 | Inciting activities not reported | |
| Football injuries on synthetic turf fields | S. Akkaya; M. Serinken; N. Akkaya; I. Turkcuer; E. Uyanik | 2011 | Inciting activities not reported | |
| Foul play is associated with injury incidence: an epidemiological study of three FIFA World Cups (2002-2010) | J. Ryynanen; A. Junge; J. Dvorak; L. Peterson; H. Kautiainen; J. Karlsson; M. Borjesson | 2013 | Inciting activities not reported | |
| Gender differences in sport injury risk and types of inju-ries: a retrospective twelve-month study on cross-country skiers, swimmers, long-distance runners and soccer players | L. Ristolainen; A. Heinonen; B. Waller; U. M. Kujala; J. A. Kettunen | 2009 | Inciting activities not reported | |
| Gender-specific differences in school sports injuries | J. Kelm; F. Ahlhelm; K. Anagnostakos; W. Pitsch; E. Schmitt; T. Regitz; D. Pape | 2004 | Mixed population | |
| Gradual increase in the risk of match injury in Norwegian male professional football: A 6-year prospective study | J. Bjorneboe; R. Bahr; T. E. Andersen | 2014 | Inciting activities not reported | |
| Harmful association of sprinting with muscle injury occurrence in professional soccer match-play: A two-season, league wide exploratory investigation from the Qatar Stars League | W. Gregson; V. Di Salvo; M. C. Varley; M. Modonutti; A. Belli; K. Chamari; M. Weston; L. Lolli; C. Eirale | 2020 | Inciting activities not reported | |
| High school football injuries: field conditions and other factors | B. L. Andresen; M. D. Hoffman; L. W. Barton | 1989 | Unable to access | |
| Illness and Injuries in Elite Football Players-A Prospective Cohort Study During the FIFA Confederations Cup 2009 | N. Theron; M. Schwellnus; W. Derman; J. Drovak | 2013 | Inciting activities not reported | |
| Impairment of Sprint Mechanical Properties in an Actual Soccer Match: A Pilot Study | R. Nagahara; J. B. Morin; M. Koido | 2016 | Did not analyse injury | |
| Incidence and characteristics of injuries during the 2011 West Africa Football Union (WAFU) Nations' Cup | A. K. Akodu; O. B. Owoeye; M. Ajenifuja; S. R. Akinbo; F. Olatona; O. Ogunkunle | 2012 | Inciting activities not reported | |
| Incidence and Descriptive Epidemiology of Injuries to College Ultimate Players | D. I. Swedler; J. M. Nuwer; A. Nazarov; S. C. Huo; L. Malevanchik | 2015 | Other sport | |
| Incidence and factors associated with injuries among adolescent players in an amateur soccer tournament in Nigeria | A. Olumide; K. Ajide | 2016 | Inciting activities not reported | |
| Incidence and Mechanisms of Collegiate Men's Soccer Match Injuries on Artificial Turf Versus Natural Grass | M. C. Meyers | 2011 | Duplicate | |
| Incidence and risk factors for injuries to the anterior cruciate ligament in National Collegiate Athletic Association football: data from the 2004-2005 through 2008-2009 National Collegiate Athletic Association Injury Surveillance System | J. L. Dragoo; H. J. Braun; J. L. Durham; M. R. Chen; A. H. Harris | 2012 | Other sport | |
| Incidence of Football and Futsal Injuries Among Youth in Malaysian Games 2018 | A. H. Ahmad-Shushami; S. Abdul-Karim | 2020 | Inciting activities not reported | |
| Incidence of football injuries in youth players - Comparison of players from two European regions | A. Junge; J. Chomiak; J. Dvorak | 2000 | Inciting activities not reported | |
| Incidence of Popular Ball Game Injuries in Indian Pre and Post Adolescent Boys | J. Sen | 2005 | Mixed population | |
| Injuries among spanish male amateur soccer players: a retrospective population study | H. Herrero; J. J. Salinero; J. Del Coso | 2014 | Inciting activities not reported | |
| Injuries and illnesses of football players during the 2010 FIFA World Cup | J. Dvorak; A. Junge; W. Derman; M. Schwellnus | 2011 | Inciting activities not reported | |
| Injuries in amateur soccer players on artificial turf: A one-season prospective study | P. Sousa; A. Rebelo; J. Brito | 2013 | Inciting activities not reported | |
| Injuries in Austrian football players: Are they an issue? | F. Fischer; C. Hoser; C. Blank; W. Schobersberger; C. Hepperger; P. Gfoller; C. Fink | 2019 | Other sport | |
| Injuries in elite youth football players: a prospective three-year study | M. Ergun; H. N. Denerel; M. S. Binnet; K. A. Ertat | 2013 | Inciting activities not reported | |
| Injuries in elite-level women's football-a two-year prospective study in the Irish Women's National League | D. Horan; C. Blake; M. Hägglund; S. Kelly; M. Roe; E. Delahunt | 2021 | Inciting activities not reported |  |
| Injuries in female football players in top-level international tournaments | A. Junge; J. Dvorak | 2007 | Inciting activities not reported | |
| Injuries in football: risk factors, injury mechanisms, team performance and prevention | A. Arnason | 2004 | Data used in another study included | |
| Injuries in formal and informal non-professional soccer - an overview of injury context, causes, and characteristics | A. Gebert; M. Gerber; U. Puhse; P. Gassmann; H. Stamm; M. Lamprecht | 2018 | Recreational participants | |
| Injuries in greek amateur soccer players | G. Tsiganos; D. Sotiropoulos; P. Baltopoulos | 2007 | Inciting activities not reported | |
| Injuries in Japanese Junior Soccer Players During Games and Practices | K. Kenji; S. Masashi; U. Ryo | 2017 | Inciting activities not reported | |
| Injuries in Portuguese Amateur Youth Football Players: A Six Month Prospective Descriptive Study | M. Nogueira; R. Laiginhas; J. Ramos; O. Costa | 2017 | Inciting activities not reported | |
| Injuries in Portuguese Youth Soccer Players During Training and Match Play | J. Brito; R. M. Malina; A. Seabra; J. L. Massada; J. M. Soares; P. Krustrup; A. Rebelo | 2012 | Inciting activities not reported | |
| Injuries in professional male football players in Kosovo: a descriptive epidemiological study | I. Shalaj; F. Tishukaj; N. Bachl; H. Tschan; B. Wessner; R. Csapo | 2016 | Inciting activities not reported | |
| Injuries in soccer | W. C. McMaster; M. Walter | 1978 | Inciting activities not reported | |
| Injuries in Spanish female soccer players | J. Del Coso; H. Herrero; J. J. Salinero | 2018 | Inciting activities not reported | |
| Injuries of veteran football (soccer) players in Germany | D. Hammes; K. Aus Der Funten; S. Kaiser; E. Frisen; J. Dvorak; T. Meyer | 2015 | Inciting activities not reported | |
| Injury characteristics in the German professional male soccer leagues after a shortened winter break | K. aus der Funten; O. Faude; J. Lensch; T. Meyer | 2014 | Inciting activities not reported | |
| Injury epidemiology in a national football team of the Middle East | C. Eirale; B. Hamilton; G. Bisciotti; J. Grantham; H. Chalabi | 2012 | Inciting activities not reported | |
| Injury evaluation of the Turkish national football team over six consecutive seasons | B. Bayraktar; C. Dinc; I. Yucesir; A. Evin | 2011 | Inciting activities not reported | |
| Injury in elite women soccer and national women soccer in the lower extremity | E. Blasco; A. Paredes; C. Monleon; M. Martin; M. Fargueta; L. Elvira | 2018 | Inciting activities not reported | |
| Injury incidence and injury patterns in professional football: the UEFA injury study | J. Ekstrand; M. Hagglund; M. Walden | 2011 | Inciting activities not reported | |
| Injury incidence in a spanish sub-elite professional football team: a prospective study during four consecutive seasons | J. Mallo; P. Gonzalez; S. Veiga; E. Navarro | 2011 | Inciting activities not reported | |
| Injury mechanisms in a Mexican soccer team | S. Echegoyen; C. Rodriguez | 1998 | Abstract only | |
| Injury patterns in professional arena football | M. A. Herbenick; J. S. King; G. Altobelli; B. Nguyen; L. Podesta | 2008 | Other sport | |
| Injury patterns in selected high school sports: a review of the 1995-1997 seasons | J. W. Powell; K. D. Barber-Foss | 1999 | Inciting activities not reported | |
| Injury prevalence, types and anotomical localizations in elite football players | T. C. Akalin; M. Goktepe; M. Gumus; K. Gokdemir; M. E. Ciplak; B. Emektar | 2020 | Inciting activities not reported |  |
| Injury Prevalence, Types and Mechanisms in Football: A Media-based Approach | R. M. Musa; I. Hassan; M. R. Abdullah; M. N. L. Azmi; S. M. Mat-Rasid | 2019 | Inciting activities not reported | |
| Injury Profile in a Brazilian First-Division Youth Soccer Team: A Prospective Study | L. G. Cezarino; B. L. da Silva Grüninger; R. S. Silva | 2020 | Inciting activities not reported | |
| Injury Profile in a Brazilian First-Division Youth Soccer Team: A Prospective Study | L. G. Cezarino; B. L. D. Gruninger; R. S. Silva | 2020 | Inciting activities not reported | |
| Injury Profile in Swedish Elite Floorball | U. Tranaeus; E. Gotesson; S. Werner | 2016 | Other sport | |
| Injury Profile of Elite Male Young Soccer Players in a Spanish Professional Soccer Club: A Prospective Study During 4 Consecutive Seasons | J. Raya-Gonzalez; L. Suarez-Arrones; A. Navandar; C. Balsalobre-Fernandez; E. Saez de Villarreal | 2019 | Inciting activities not reported | |
| Injury Profile of Elite Male Young Soccer Players in a Spanish Professional Soccer Club: A Prospective Study During 4 Consecutive Seasons | J. Raya-González; L. Suárez-Arrones; A. Navandar; C. Balsalobre-Fernández; E. Sáez de Villarreal | 2020 | Inciting activities not reported | |
| Injury risk for goalkeepers in norwegian male professional football | E. Strand; T. Krosshaug; T. E. Andersen | 2011 | Abstract only | |
| Injury surveillance during a 2-day national female youth football tournament in Kenya | M. Lislevand; T. E. Andersen; A. Junge; J. Dvorak; K. Steffen | 2014 | Inciting activities not reported | |
| Injury surveillance during a national female youth football tournament in kenya | M. Lislevand; K. Steffen; A. Junge; J. Dvorak; T. E. Andersen | 2011 | Abstract only | |
| Injury Surveillance in Major League Soccer: A 4-Year Comparison of Injury on Natural Grass Versus Artificial Turf Field | S. P. Calloway; D. M. Hardin; M. D. Crawford; J. M. Hardin; L. J. Lemak; E. Giza; B. Forsythe; Y. Lu; B. H. Patel; D. C. Osbahr; M. B. Gerhardt; B. R. Mandelbaum; W. W. Baldwin | 2019 | Inciting activities not reported | |
| Injury surveillance in the World Football Tournaments 1998-2012 | A. Junge; J. Dvorak | 2013 | Inciting activities not reported | |
| Injury surveillance survey results from the First Annual Gulf Council Sports Medicine Conference, Abu Dhabi, UAE | J. A. Nyland; B. D. Stocker; D. N. M. Caborn; B. Adkisson; J. A. Brosky | 1997 | Mixed population | |
| Le lesioni nel calcio. Epidemiologia e meccanismi. / The epidemiology and mechanisms of soccer injuries | F. Latella; G. Serni; P. Aglietti; G. Zaccherotti; P. De Biase | 1992 | Unable to access | |
| Lunacy revisited - the myth of the full moon: are football injuries related to the lunar cycle? | N. Yousfi; R. N. Rekik; C. Eirale; R. Whiteley; A. Farooq; M. Tabben; S. Gillogly; R. Bahr; K. Chamari | 2018 | Inciting activities not reported | |
| Mechanisms of injury of the anterior cruciate ligament in soccer players | A. J. Delfico; W. E. Garrett, Jr. | 1998 | Review | |
| Moderate to severe injuries in football: a one-year prospective study of twenty-four female and male amateur teams | A. Lion; D. Theisen; T. Windal; L. Malisoux; C. Nuhrenborger; R. Huberty; A. Urhausen; R. Seil | 2014 | Inciting activities not reported | |
| Muscle injuries in the academy of a Spanish professional football club: A one-year prospective study | J. Raya-Gonzalez; L. Suarez-Arrones; J. Larruskain; E. S. de Villarreal | 2018 | Inciting activities not reported | |
| Natural and synthetic grass. A comparative study on the incidence of muscle injuries for senior athletes | M. Pietro | 2019 | Inciting activities not reported | |
| Orthopaedics injuries in male professional football players in Brazil: a prospective comparison between two divisions | G. G. Arliani; P. H. S. Lara; D. C. Astur; A. Pedrinelli; J. R. Pagura; M. Cohen | 2017 | Inciting activities not reported | |
| Orthopedic injuries in a formation of a soccer club | D. A. Carvalho | 2013 | Inciting activities not reported | |
| Orthopedic injuries in men's professional soccer in brazil: prospective comparison of two consecutive seasons 2017/2016 | E. R. de Moraes; G. G. Arliani; P. H. S. Lara; E. H. R. da Silva; J. R. Pagura; M. Cohen | 2018 | Inciting activities not reported | |
| Physical fitness, injuries, and team performance in soccer | A. Arnason; S. B. Sigurdsson; A. Gudmundsson; I. Holme; L. Engebretsen; R. Bahr | 2004 | Inciting activities not reported | |
| Predictors of moderate and severe injuries in Italian major leagues soccer teams: results from a cohort study | S. Raimonde; E. Taioli | 2007 | Inciting activities not reported | |
| Pre-season Fitness Level and Injury Rate in Professional Soccer - A Prospective Study | E. Eliakim; O. Doron; Y. Meckel; D. Nemet; A. Eliakim | 2018 | Inciting activities not reported | |
| Pre-season injuries in Scottish football: a prospective study | G. M. Mackay; W. S. Hillis | 1996 | Unable to access | |
| Prospective Evaluation of Injuries occurred during the Brazilian Soccer Championship in 2016 | D. C. Netto; G. G. Arliani; E. S. Thiele; M. N. L. Cat; M. Cohen; J. R. Pagura | 2019 | Written in other language | |
| Prospective evaluation of injuries ocurred during a professional soccer championship in 2016 in sao paulo, brazil | G. G. Arliani; P. H. S. Lara; D. C. Astur; A. Pedrinelli; J. R. Pagura; M. Cohen | 2017 | Inciting activities not reported | |
| Rapid Posterior Tibial Reduction After Noncontact Anterior Cruciate Ligament Rupture: Mechanism Description From a Video Analysis | A. Grassi; F. Tosarelli; P. Agostinone; L. Macchiarola; S. Zaffagnini; F. Della Villa | 2020 | Inciting activities not reported | |
| Risk assessment in professional football: an examination of accidents and incidents in the 1994 World Cup finals | R. D. Hawkins; C. W. Fuller | 1996 | Inciting activities not reported | |
| Risk factors for injuries in elite female soccer players | O. Faude; A. Junge; W. Kindermann; J. Dvorak | 2006 | Inciting activities not reported | |
| Risk of injury on third-generation artificial turf in Norwegian professional football | J. Bjorneboe; R. Bahr; T. E. Andersen | 2010 | Inciting activities not reported | |
| Safety of third-generation artificial turf in male elite professional soccer players in Italian major league | R. M. Lanzetti; A. Ciompi; D. Lupariello; M. Guzzini; A. De Carli; A. Ferretti | 2017 | Inciting activities not reported | |
| Saudi Professional League: A Prospective Study of the Injuries and Illnesses Sustained by Professional Soccer Players During the 2015 - 2016 Season | Q. I. Muaidi | 2019 | Inciting activities not reported | |
| Soccer Injuries and Recovery in Dutch Male Amateur Soccer Players: Results of a Prospective Cohort Study | A.-M. van Beijsterveldt; K. Steffen; J. H. Stubbe; J. E. Frederiks; I. G. L. van de Port; F. J. G. Backx | 2014 | Inciting activities not reported | |
| Soccer injury in the lower extremities | P. Wong; Y. Hong | 2005 | Review | |
| Sports injuries profile of a first division Brazilian soccer team: a descriptive cohort study | G. F. Reis; T. R. Santos; R. C. Lasmar; O. Oliveira Junior; R. F. Lopes; S. T. Fonseca | 2015 | Inciting activities not reported | |
| Tackle mechanisms and match characteristics in women's elite football tournaments | P. Tscholl; D. O'Riordan; C. W. Fuller; J. Dvorak; A. Junge | 2007 | Did not analyse injury | |
| Ten-Year Epidemiology of Ankle Injuries in Men's and Women's Collegiate Soccer Players | M. Gulbrandsen; D. E. Hartigan; K. A. Patel; J. L. Makovicka; S. V. Tummala; A. Chhabra | 2019 | Inciting activities not reported | |
| The epidemiology of ankle injuries occurring in English Football Association academies | D. J. Cloke; S. Spencer; A. Hodson; D. Deehan | 2009 | Mixed population | |
| The epidemiology of anterior cruciate ligament injury in football (soccer): a review of the literature from a gender-related perspective | M. Walden; M. Hagglund; J. Werner; J. Ekstrand | 2011 | Review | |
| The Epidemiology of Injuries in Middle School Football, 2015-2017: The Advancing Healthcare Initiatives for Underserved Students Project | Z. Y. Kerr; N. Cortes; J. P. Ambegaonkar; A. M. Caswell; M. Prebble; K. Romm; S. V. Caswell | 2019 | Other sport | |
| The incidence and nature of injuries sustained on grass and 3rd generation artificial turf: a pilot study in elite Saudi National Team footballers | M. Almutawa; M. Scott; K. P. George; B. Drust | 2014 | Inciting activities not reported | |
| The influence of match frequency on the risk of injury in professional soccer | J. Vilamitjana; N. Lentini; E. Masabeu | 2013 | Inciting activities not reported | |
| Time-trends and Inciting activities surrounding ankle injuries in men's professional football: an 11-year follow-up of the UEFA Champions League injury study | M. Walden; M. Hagglund; J. Ekstrand | 2013 | Inciting activities not reported | |
| Training habits and injuries of masters' level football players: A preliminary report | R. Newsham-West; C. Button; P. D. Milburn; A. Mundermann; G. Sole; A. G. Schneiders; S. J. Sullivan | 2009 | Inciting activities not reported | |
| UEFA injury study--an injury audit of European Championships 2006 to 2008 | M. Hagglund; M. Walden; J. Ekstrand | 2009 | Inciting activities not reported | |
| Video analysis of causes and mechanism of the ACL injuries in the Iranian professional soccer player | H. Tarmah; N. Rahnama; K. Khayyambashi | 2010 | Abstract only | |

Table S5 PRISMA 2020 Checklist

| **Section and Topic** | **Item #** | **Checklist item** | **Location where item is reported** |
| --- | --- | --- | --- |
| **TITLE** | | |  |
| Title | 1 | Identify the report as a systematic review. | Title |
| **ABSTRACT** | | |  |
| Abstract | 2 | See the PRISMA 2020 for Abstracts checklist. | Abstract |
| **INTRODUCTION** | | |  |
| Rationale | 3 | Describe the rationale for the review in the context of existing knowledge. | Page 2 |
| Objectives | 4 | Provide an explicit statement of the objective(s) or question(s) the review addresses. | Page 2 |
| **METHODS** | | |  |
| Eligibility criteria | 5 | Specify the inclusion and exclusion criteria for the review and how studies were grouped for the syntheses. | Page 2 |
| Information sources | 6 | Specify all databases, registers, websites, organisations, reference lists and other sources searched or consulted to identify studies. Specify the date when each source was last searched or consulted. | Page 2-3 |
| Search strategy | 7 | Present the full search strategies for all databases, registers and websites, including any filters and limits used. | Table S1 |
| Selection process | 8 | Specify the methods used to decide whether a study met the inclusion criteria of the review, including how many reviewers screened each record and each report retrieved, whether they worked independently, and if applicable, details of automation tools used in the process. | Page 3 |
| Data collection process | 9 | Specify the methods used to collect data from reports, including how many reviewers collected data from each report, whether they worked independently, any processes for obtaining or confirming data from study investigators, and if applicable, details of automation tools used in the process. | Page 3-5 |
| Data items | 10a | List and define all outcomes for which data were sought. Specify whether all results that were compatible with each outcome domain in each study were sought (e.g. for all measures, time points, analyses), and if not, the methods used to decide which results to collect. | Page 3-5 |
|  | 10b | List and define all other variables for which data were sought (e.g. participant and intervention characteristics, funding sources). Describe any assumptions made about any missing or unclear information. | Page 3-5 |
| Study risk of bias assessment | 11 | Specify the methods used to assess risk of bias in the included studies, including details of the tool(s) used, how many reviewers assessed each study and whether they worked independently, and if applicable, details of automation tools used in the process. | Page 3-5 |
| Effect measures | 12 | Specify for each outcome the effect measure(s) (e.g. risk ratio, mean difference) used in the synthesis or presentation of results. | Page 5 |
| Synthesis methods | 13a | Describe the processes used to decide which studies were eligible for each synthesis (e.g. tabulating the study intervention characteristics and comparing against the planned groups for each synthesis (item #5)). | NA |
|  | 13b | Describe any methods required to prepare the data for presentation or synthesis, such as handling of missing summary statistics, or data conversions. | Page 3-5 |
|  | 13c | Describe any methods used to tabulate or visually display results of individual studies and syntheses. | Page 5 |
|  | 13d | Describe any methods used to synthesize results and provide a rationale for the choice(s). If meta-analysis was performed, describe the model(s), method(s) to identify the presence and extent of statistical heterogeneity, and software package(s) used. | Page 3-5 |
|  | 13e | Describe any methods used to explore possible causes of heterogeneity among study results (e.g. subgroup analysis, meta-regression). | NA |
|  | 13f | Describe any sensitivity analyses conducted to assess robustness of the synthesized results. | NA |
| Reporting bias assessment | 14 | Describe any methods used to assess risk of bias due to missing results in a synthesis (arising from reporting biases). | NA |
| Certainty assessment | 15 | Describe any methods used to assess certainty (or confidence) in the body of evidence for an outcome. | NA |
| **RESULTS** | | |  |
| Study selection | 16a | Describe the results of the search and selection process, from the number of records identified in the search to the number of studies included in the review, ideally using a flow diagram. | Page 5 |
|  | 16b | Cite studies that might appear to meet the inclusion criteria, but which were excluded, and explain why they were excluded. | Table S4 |
| Study characteristics | 17 | Cite each included study and present its characteristics. | Table 2-3, Table S6 |
| Risk of bias in studies | 18 | Present assessments of risk of bias for each included study. | Supplementary File |
| Results of individual studies | 19 | For all outcomes, present, for each study: (a) summary statistics for each group (where appropriate) and (b) an effect estimate and its precision (e.g. confidence/credible interval), ideally using structured tables or plots. | NA |
| Results of syntheses | 20a | For each synthesis, briefly summarise the characteristics and risk of bias among contributing studies. | Page 15 and 18, Fig. 6 and S3 |
|  | 20b | Present results of all statistical syntheses conducted. If meta-analysis was done, present for each the summary estimate and its precision (e.g. confidence/credible interval) and measures of statistical heterogeneity. If comparing groups, describe the direction of the effect. | NA |
|  | 20c | Present results of all investigations of possible causes of heterogeneity among study results. | NA |
|  | 20d | Present results of all sensitivity analyses conducted to assess the robustness of the synthesized results. | NA |
| Reporting biases | 21 | Present assessments of risk of bias due to missing results (arising from reporting biases) for each synthesis assessed. | NA |
| Certainty of evidence | 22 | Present assessments of certainty (or confidence) in the body of evidence for each outcome assessed. | NA |
| **DISCUSSION** | | |  |
| Discussion | 23a | Provide a general interpretation of the results in the context of other evidence. | Page 17-20 |
|  | 23b | Discuss any limitations of the evidence included in the review. | Page 20-21 |
|  | 23c | Discuss any limitations of the review processes used. | Page 21-22 |
|  | 23d | Discuss implications of the results for practice, policy, and future research. | Page 22 |
| **OTHER INFORMATION** | | |  |
| Registration and protocol | 24a | Provide registration information for the review, including register name and registration number, or state that the review was not registered. | Abstract and page 2 |
|  | 24b | Indicate where the review protocol can be accessed, or state that a protocol was not prepared. | Page 2 |
|  | 24c | Describe and explain any amendments to information provided at registration or in the protocol. | Page 2 |
| Support | 25 | Describe sources of financial or non-financial support for the review, and the role of the funders or sponsors in the review. | Declarations |
| Competing interests | 26 | Declare any competing interests of review authors. | Declarations |
| Availability of data, code and other materials | 27 | Report which of the following are publicly available and where they can be found: template data collection forms; data extracted from included studies; data used for all analyses; analytic code; any other materials used in the review. | Declarations |

*From:*  Page MJ, McKenzie JE, Bossuyt PM, Boutron I, Hoffmann TC, Mulrow CD, et al. The PRISMA 2020 statement: an updated guideline for reporting systematic reviews. BMJ 2021;372:n71. doi: 10.1136/bmj.n71. For more information, visit: http://www.prisma-statement.org/

Table S6 Information of studies reporting inciting activities leading to overall injuries

| **Study** | **Sex** | **Age** | **Competitive level** | **Main aim** | **Tool** | **Injuries analysed (N)** | **Reported injury definition** | **Phase of play** | **Player location** | **Player activity (M-F)** |
| --- | --- | --- | --- | --- | --- | --- | --- | --- | --- | --- |
| Zeren and Oztekin [120] | M & F | 24 | Professional | Other | Interview | 9 | No | Score-celebration: 100% | Not reported | Sliding: 56%  Piling up: 33%  Racing away: 11% |
| Kittipong and Arth Na [121] | F | 19.96 ± 2.23 | Elite | Inciting activity analysis | Questionnaire | 210 | No | Not reported | Not reported | Collision: 16%  Contact with floor: 4%  Foul: 6%  Kicked: 27%  Tackled: 17%  Kicking: 8%  Overuse: 7%  Running: 10% Other*: 6 |
| Bastos et al. [111] | M | 14.67 ± 2.08 | Not stated | Inciting activity analysis | Questionnaires | 56 | Yes, supported by non-consensus reference | Not reported | Not reported | Impact: 57%  Jumping: 14%  Running: 33%  Specific action: 25% |
| Hawkins et al. [114] | M | Adults | Professional | Other | Questionnaires | 6030 | Yes, not supported by any reference | Not reported | Not reported | Collision: 6%  Kicked: 5%  Tackled: 15%  Tackling: 9%  Use of elbow: 1%  Diving: 1%  Falling: 1%  Heading: 1%  Jumping: 2%  Landing: 4%  Passing: 4%  Running: 19%  Shooting: 4%  Stretching: 6%  Twisting/turning: 8% Other*: 14 |
| Jacobs and Van Den Berg [113] | M | 16.2 ± 1.13 | Elite | Inciting activity analysis | Questionnaires | 544 | No | Not reported | Not reported | Collision: 3%  Tackled: 8%  Tackling: 4%  Heading: 1%  Jumping: 3%  Landing: 3%  Running: 6%  Shooting: 6%  Twisting/turning: 3% Other*: 64% |
| Sentsomedi and Puckree [117] | F | 15.85 ± 1.32 | High school | Other | Questionnaires | 80 | No | Not reported | Not reported | Collision: 18%  Tackling/being tackled: 14%  Burns: 5%  Heading: 8%  Jumping: 4%  Kicking: 6%  Landing: 14%  Running: 19%  Shooting: 19%  Turning: 6% |
| Woods et al. [122] | M | Adults* | Professional | Other | Questionnaires | 6030 | Yes, not supported by any reference | Not reported | Not reported | Collision: 6%  Kicked: 5%  Tackled: 15%  Tackling: 9%  Use of elbow: 1%  Diving: 1%  Falling: 1%  Heading: 1%  Jumping: 2%  Landing: 4%  Passing: 4%  Running: 19%  Shooting: 4%  Stretching: 6%  Twisting/turning: 8% Other*: 14% |
| Azubuike [116] | M | 20 | Professional and amateur | Other | Questionnaires + interview | 204 | No | Not reported | Not reported | Collision: 2%  Tackling/being tackled: 45%  Jumping/landing: 12%  Running: 7%  Shooting: 9%  Twisting/turning: 11% Other*: 15% |
| Arnason et al. [123] | M | 25, range 18-34 | Elite | Other | Report | 129 | Yes, supported by consensus reference | Not reported | Not reported | Tackling: 16%  Cutting: 4%  Overuse: 6%  Shooting/kicking: 9%  Sprinting: 11% Other*: 54% |
| Carling et al. [115] | M | Adults* | Elite | Inciting activity analysis | Report | 10 | Unclear | Not reported | Not reported | Duel: 40%  Tackling: 10%  Ball handling/controlling: 10%  Jumping: 10%  Landing: 10%  Passing: 10%  Running: 20%  Sprinting: 80% |
| Chandran et al. [119] | M | Collegiate | College | Other | Report | 2821 | Yes, not supported by any reference | Not reported | Not reported | On the ball: 2% Blocking shot: 3% Conditioning: 2% Set pieces: 2% Defending: 11% General play: 32% Goalkeeping: 5% Heading: 5% Loose ball: 3% Passing: 3% Receiving: 2% Running: 11% Finishing: 4% Slide tackling: 5% Other*: 10% |
| Chandran et al. [124] | F | Collegiate | College | Other | Report | 3932 | Yes, not supported by any reference | Not reported | Not reported | On the ball: 2% Blocking shot: 3% Conditioning: 2% Set pieces: 2% Defending: 10% General play: 33% Goalkeeping: 6% Heading: 4% Loose ball: 3% Passing: 2% Receiving: 1% Running: 11% Finishing: 4% Slide tackling: 5% Other*: 11% |
| de Freitas Guina Fachina et al. [108] | M | 25.7 ± 4.3 | Professional | Inciting activity analysis | Report | 95 | Yes, not supported by any reference | Not reported | Not reported | Collision: 24%  Decelerating: 5%  Dribbling: 1%  Falling: 4%  Heading: 1%  Jumping: 8%  Kicking: 13%  Lateral movements: 2%  Passing: 6%  Running: 16% Other*: 19% |
| DiStefano et al. [125] | F | High school and college | High school and college | Other | Report | 8051 | Yes, not supported by any reference | Not reported | Not reported | Tackled: 10%  Tackling: 5%  Ball handling/controlling: 1%  Blocking a shot or pass: 2%  Conditioning: 11%  Chasing a loose ball: 8%  Defending: 12%  Diving: 6%  General play: 31%  Heading: 5%  Passing: 5%  Receiving: 1%  Shooting: 3% |
| Ekstrand and Gillquist [126] | M | 24.6 ± 4.6 | Not stated | Inciting activity analysis | Report | 256 | Yes, not supported by any reference | Not reported | Not reported | Most contact injuries were caused by tackling or kicking, and most non-contact injuries occurred during running or cutting |
| Faude et al. [35] | F | 22.4 ± 5.0 | Professional | Other | Report | 241 | Yes, supported by consensus reference | Not reported | Not reported | Collision: 3%  Contact with ball: 4%  Foul: 10%  Tackled: 15%  Tackling: 15%  Changing direction: 9%  Jumping: 5%  Shooting: 12%  Slipping: 6% Other*: 18% |
| Fitzharris et al. [29] | M | 23 ± 4.7 | Semi professional | Other | Report | 152 | Yes, supported by consensus reference | Not reported | Not reported | Collision: 22%  Contact ball: 1%  Tackling: 11%  Diving: 1%  Falling: 1%  Kicking: 1%  Landing: 7%  Running: 31%  Stretching: 6%  Twisting/turning: 8% Other*: 11% |
| Gaulrapp et al. [27] | F | 22.8, range 16-35 | Professional | Inciting activity analysis | Report | 246 | Yes, supported by consensus reference | Not reported | Not reported | Tackled: 9%  Tackling: 26%  Falling: 4%  Fatigue: 24%  Twisting: 4%  Turf: 4% Other*: 29% |
| Hassabi et al. [118] | M | 24 ± 3.0 | Professional | Inciting activity analysis | Report | 50 | Yes, not supported by any reference | Not reported | Defensive third: 14%  Mid-field third: 2%  Offensive third: 8% | Contact: 44%  Ball contact: 4%  Diving: 1%  Falling: 3%  Overuse: 4%  Running or other individual activities: 14% Other*: 30% |
| Hawkins and Fuller [127] | M | Adults* | Professional | Other | Report | 744 | Yes, not supported by any reference | Not reported | Not reported | Collision: 4%  Tackled: 23%  Tackling: 14%  Heading: 2%  Jumping: 2%  Landing: 5%  Overuse / Growth: 8%  Running: 19%  Shooting: 10%  Twisting/turning: 8% Other*: 5% |
| Ibikunle et al. [128] | F | 21.80 ± 4.55 | Professional | Inciting activity analysis | Report | 62 | Yes, not supported by any reference | Not reported | Not reported | Collision: 10%  Contact ball: 3%  Kicked: 11%  Tackled: 15%  Tackling: 3%  Diving: 3%  Dribbling: 6%  Heading: 5%  Jumping/landing: 6%  Running: 6%  Shooting: 5%  Slipping: 10%  Stretching: 6%  Twisting/turning: 8% Other*: 3% |
| Kerr et al. [129] | M | High school and college | Not stated | Other | Report | 7677 | Yes, not supported by any reference | Not reported | Not reported | Tackled: 3%  Tackling: 2%  Ball handling/controlling: 9%  Blocking a shot or pass: 2%  Conditioning: 4%  Chasing a loose ball: 8%  Defending: 11%  Diving: 7%  General play: 31%  Heading: 7%  Passing: 5%  Receiving: 3%  Shooting: 6% |
| Khodaee et al. [110] | M & F | High school* | Not stated | Inciting activity analysis | Report | 6154 | Yes, not supported by any reference | Not reported | Def middle: 13% - 13%  Def side: 9% - 8%  Def midfield: 32% - 37%  Off midfield: 19% – 19%  Off middle: 8% - 12%  Off side: 9% - 8% | Contact ball: 11% - 14%  Kicked: 10% - 10%  Tackled: 2% - 2%  Tackling: 6% - 4%  Blocking a shot or pass: 2% - 2%  Loose ball: 12% - 11%  Conditioning: 4% - 5%  Defending: 10% - 16%  Diving: 8% - 6%  Dribbling: 11% - 10%  General play: 24% - 24%  Heading: 9% - 6%  Passing: 5% - 6%  Receiving: 5% - 4%  Shooting: 5% - 5% |
| Meyers [109] | M | Collegiate | Collegiate | Inciting activity analysis | Report | 722 | Yes, supported by non-consensus reference | Warmup: 4%  Offensive direct play: 29%  Possession buildup: 13%  Offensive counterattack: 7%  Defensive high pressure: 21%  Defensive middle pressure: 13%  Defensive low pressure: 6%  Kicks penalty/corner/indirect: 7% | Not reported | Contact floor: 7%  Stepped on/fallen/kicked: 9%  Tackled: 19%  Tackling: 5%  Blocking a shot or pass: 4%  Chasing a loose ball: 8%  Diving: 4%  Dribbling/shielding: 7%  Heading: 8%  Heat illness: 1%  Non-contact rotation/jump/sprint: 12%  Overuse: 3%  Passing/receiving pass: 9%  Shooting: 4% |
| Meyers [106] | F | Collegiate | Not stated | Inciting activity analysis | Report | 693 | Yes, supported by non-consensus reference | Kickoff: 3%  Offensive direct play: 0  Possession buildup: 21%  Breakdown: 15%  Elaborate actions: 10%  Defensive middle pressure: 23%  Defensive low pressure: 14%  Set play: 6%  Ineffective attack: 7% | Not reported | Contact floor: 14%  Stepped on/fallen/kicked: 9%  Tackled: 17%  Tackling: 4%  Blocking a shot or a pass: 6%  Chasing a loose ball: 8%  Diving: 4%  Dribbling/shielding: 8%  Heading: 8%  Heat illness: 1%  Non-contact rotation/jump/sprint: 10%  Overuse: 4%  Passing/receiving pass: 4%  Shooting: 3% |
| Nilsson et al. [112] | M | 17.7, range 15-19 | Elite | Other | Report | 61 | Yes, supported by consensus reference | Not reported | Not reported | Kick/knee from opponent: 5%  Tackled: 3%  Blocking: 3%  Falling: 8%  Jumping/landing: 7%  Overload: 16%  Passing: 2%  Running/sprint: 28%  Shooting: 10%  Stretching: 3%  Twisting/turning: 2% Other*: 13% |
| Steffen et al. [130] | F | U17 | Not stated | Other | Report | 230 | Yes, supported by consensus reference | Not reported | Not reported | Collision: 8%  Tackling: 53%  Heading: 4%  Running: 21% Other*: 15% |
| Stubbe et al. [131] | M | 24.6 ± 6 4.3 | Professional | Inciting activity analysis | Report | 286 | Yes, supported by consensus reference | Not reported | Not reported | Contact with player: 33% Artificial turf: 2%  Distorting: 7%  Falling: 3%  Fatigue: 11%  Jumping: 12%  Playing field conditions: 6%  Receiving: 6%  Shooting: 6%  Twisting/turning: 8%  Weather conditions: 2% |
| Yard et al. [132] | M & F | Not reported | High school* | Other | Report | 1524 | Yes, not supported by any reference | Not reported | Not reported | Contact another player: 40% - 46%  Contact floor: 18% - 17%  Tackled: 4% - 3%  Tackling: 3% – 1%  Blocking a shot or pass: 3% – 1%  Chasing a loose ball: 12% - 14%  Conditioning: 5% - 5%  Defending: 8% – 14%  Diving: 7% – 5%  Dribbling/shielding: 14% - 14%  General play: 21% - 21%  Heading: 8% - 6%  Overuse / Growth: 6% - 5%  Passing: 6% - 6%  Receiving: 4% – 3%  Shooting: 5% - 4% |
| Drummond et al. [50] | M | 26.53 ± 4.75 | Professional | Other | Video | 92 | No | Not reported | Not reported | Run/sprint: 34%  Twist: 4%  Kick: 12% Pass/crossover: 2% Dribble: 1% Jump/landing: 6% Fall: 2% Stretching: 1% Slipping: 1% Overuse: 5% Hit by ball: 3% Collision: 2% Heading: 3% Being tackled: 2% Tackling: 1% Being kicked: 5% Blocked: 2% Other*: 14% |
| Klein et al. [60] | M | Adults* | Professional | Inciting activity analysis | Video | 345 | No | Ball possession: 52% Ball non possession: 41% | Not reported | Running: 27% Sprinting: 23% Jumping: 20% Lunging: 10% Dribbling: 16% Attacking the ball/opponent: 14% Heading: 13% Tackling: 16% Duels: 83% |
| Andersen et al. [62] | M | U21 | Professional | Inciting activity analysis | Video | 52 | Yes, not supported by any reference | Attacking phase: 71%  Defensive phase: 29% | Def midfield: 35%  Off midfield: 23%  Opponents penalty box: 13%  Defensive third: 17%  Offensive third: 12% | Screening: 2%  Tackling: 69%  Heading: 12%  Running: 2% Other*: 15% |
| Bjørneboe et al. [64] | M | Adults* | Professional | Other | Video | 1287 | Yes, supported by non-consensus reference | Not reported | Not reported | Contact another player: 88%  Contact ball: 4% Other*: 8% |
| Hawkins and Fuller [26] | M | Adults* | Professional | Other | Video | 578 | Yes, supported by non-consensus reference | Not reported | Not reported | Collision: 9%  Tackled: 18%  Tackling: 11%  Heading: 16% Other*: 45% |
| Rahnama et al. [63] | M | Adults* | Professional | Inciting activity analysis | Video | 20 | Yes, not supported by any reference | Not reported | Def middle: 5%  Def side: 5%  Def-mid middle: 20%  Def-mid side: 15%  Off-mid side: 25%  Off middle: 15%  Off side: 15% | Receiving a charge: 5%  Tackled: 70%  Tackling: 15%  Diving: 5%  Kicking: 5% |
| Tscholl et al. [61] | F | Adults and collegiate* | Professional | Other | Video | 230 | Yes, supported by consensus reference | Not reported | Not reported | Tackled: 48%  Tackling: 39% Hit by ball: 6%  Changing direction: 1%  Running: 6% Shooting: 8% Other*: 5% |
| Andersen et al. [28] | M | Adults* | Professional | Inciting activity analysis | Video, football incident analysis | 52 | Yes, supported by consensus reference | Attacking phase: 57%  Defensive phase: 42% | Def middle: 25%  Def side: 8%  Def-mid middle: 15%  Def-mid side: 13%  Off-mid middle: 8%  Off-mid side: 11%  Off middle: 2%  Off side: 9%  Off score box: 26% | Blocking: 6%  Screening: 2%  Clearing: 6%  Diving: 4%  Dribbling: 11%  Heading: 21%  Passing: 8%  Receiving: 8%  Shooting: 8% Other*: 27% |
| Arnason et al. [30] | M | Adults* | Elite | Unclear | Video, football incident analysis | 28 | Yes, supported by non-consensus reference | Breakdown: 14%  Elaborate actions: 6%  Long pass attack: 3%  Set play: 6%  Ineffective attack: 23%  Good opp defense: 17%  Attacking phase: 16%  Defensive phase: 14% | Def middle: 9%  Def side: 1%  Def-mid middle: 3%  Def-mid side: 2%  Off-mid middle: 4%  Off-mid side: 5%  Off middle: 3%  Off side: 2%  Off score boxe: 2% | Duel: 75%  Foul: 18%  Screening: 29%  Tackled: 29%  Tackling: 17%  Crossing: 7%  Deflection: 39%  Flick: 7%  Heading: 10%  Passing: 50%  Running or sprinting: 75% |

Competitive levels are reported as originally described by the studies. Participants’ age is reported as originally described in the studies. If this was not described it was either deduced by the study or, when this was not possible, it was reported as unclear and indicated in this table with an asterisk (*). Percentages are calculated dividing the total number of injuries occurred during each activity, phase of play, or location by the total number of injuries occurred. Activities reported as “Other” indicate the percentage of injuries whose activities were not reported or reported as other or unknown.


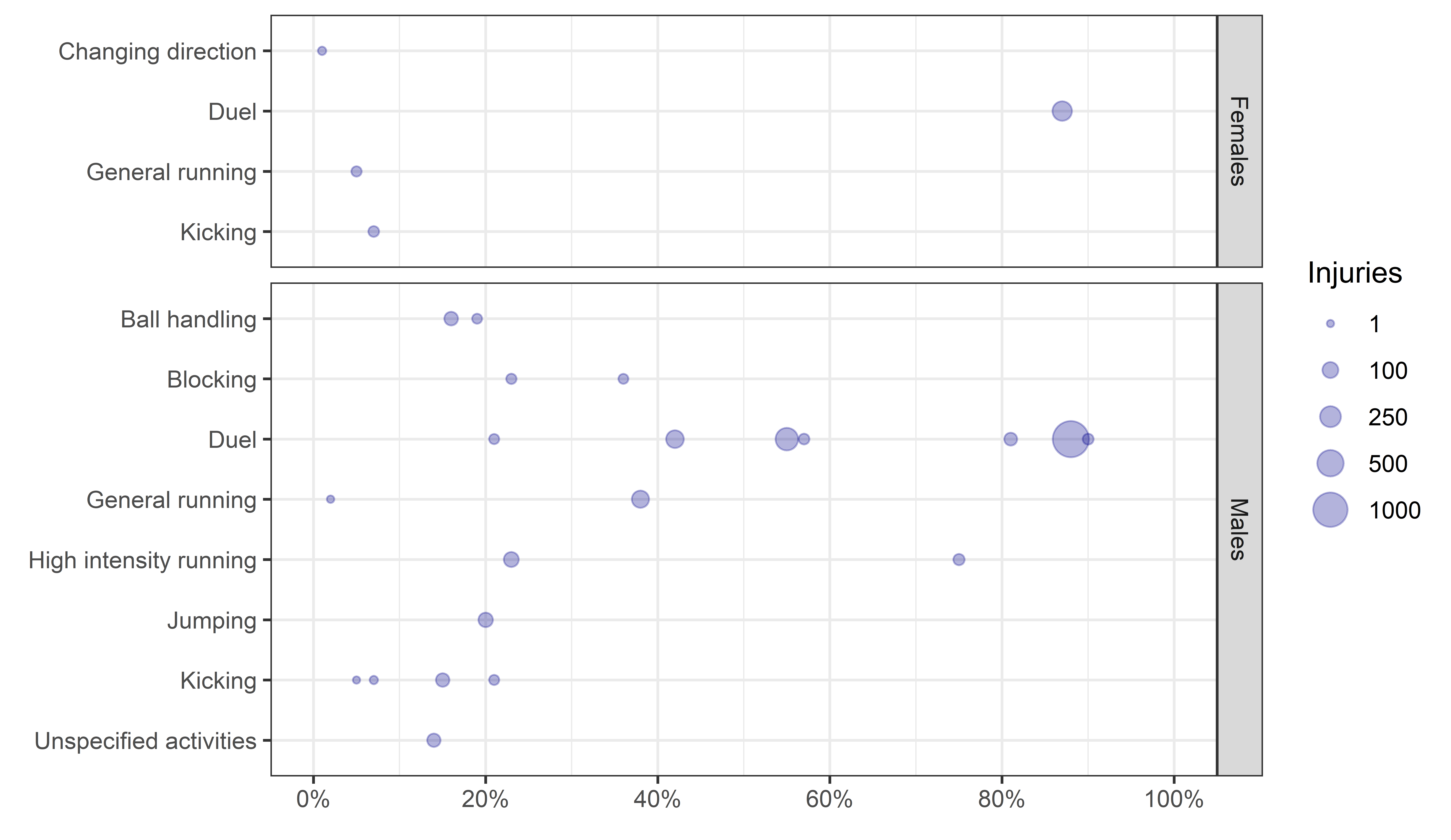


Fig. S1 Percentage of injuries occurred during specific inciting activities analysed through video-analysis by sex. Size of the dot represents the amount of injuries. Number of injuries reported: Females =293, Males = 2528


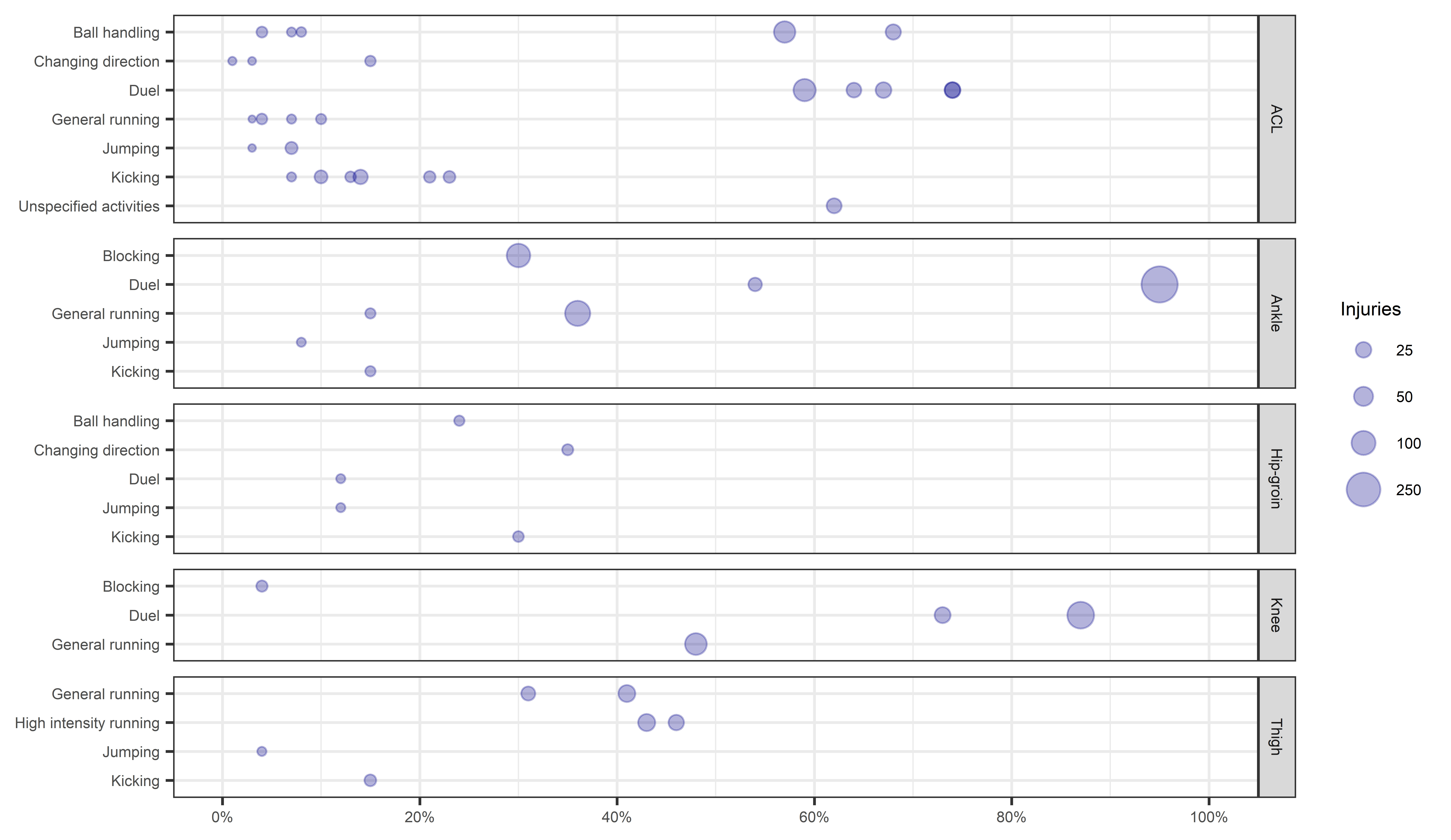


Fig. S2 Percentage of injuries occurred during specific inciting activities analysed through video-analysis by injury type. Size of the dot represents the amount of injuries. Knee injuries represent all non-ACL knee injuries. Number of injuries reported: ACL = 422, Ankle = 415, Hip-groin = 17, Knee (no ACL) = 193, Thigh = 133

Fig. S3 RoB of studies analysing different type of injuries
